# AI + Ethics Curricula for Middle School Youth: Lessons Learned from Three Project-Based Curricula

**DOI:** 10.1007/s40593-022-00298-y

**Published:** 2022-08-01

**Authors:** Randi Williams, Safinah Ali, Nisha Devasia, Daniella DiPaola, Jenna Hong, Stephen P. Kaputsos, Brian Jordan, Cynthia Breazeal

**Affiliations:** MIT Media Lab, Cambridge, MA 02142 USA

**Keywords:** Artificial Intelligence (AI), AI Literacy, Curriculum, Middle-school, Online Learning, Constructionism

## Abstract

Artificial Intelligence (AI) is revolutionizing many industries and becoming increasingly ubiquitous in everyday life. To empower children growing up with AI to navigate society’s evolving sociotechnical context, we developed three middle school AI literacy curricula: *Creative AI, Dancing with AI,* and *How to Train Your Robot.* In this paper we discuss how we leveraged three design principles—active learning, embedded ethics, and low barriers to access – to effectively engage students in learning to create and critique AI artifacts. During the summer of 2020, we recruited and trained in-service, middle school teachers from across the United States to co-instruct online workshops with students from their schools. In the workshops, a combination of hands-on unplugged and programming activities facilitated students’ understanding of AI. As students explored technical concepts in tandem with ethical ones, they developed a critical lens to better grasp how AI systems work and how they impact society. We sought to meet the specified needs of students from a range of backgrounds by minimizing the prerequisite knowledge and technology resources students needed to participate. Finally, we conclude with lessons learned and design recommendations for future AI curricula, especially for K-12 in-person and virtual learning.

## Introduction

Artificial Intelligence (AI) is the study of how humans construct machines to embody mechanisms of thought and intelligent behavior (AAAI, 2020). From social media apps to education tools to interactive voice agents, AI is becoming an increasingly prominent part of everyday life. However, members of the general public are often unaware that they are interacting with algorithms which might infringe on their privacy or contain harmful biases toward or against different demographic groups. Societal obliviousness together with technological opaqueness present serious concerns about people’s ability to safely and critically consume, use, and collaborate with AI (Long & Magerko, 2020). Traditional computer science, programming, and digital literacy skills will not be enough to successfully navigate society’s evolving socio-technical context (Touretzky et al., 2019b). Students growing up in the era of AI must be equipped with the knowledge and skills to participate in the creation and critique of AI artifacts (DiPaola et al., 2020).

The movement to teach artificial intelligence K-12 to students, which has roots in the beginning of the artificial intelligence movement (Solomon et al., 2020), has surged in recent years. Over the past five years, multiple AI literacy platforms and lesson plans have been developed for K-12 students (Long & Magerko, 2020; Touretzky et al., 2019b). Touretzky et al. (2019a) outlined recent efforts to teach AI to K-12 students, covering curricula that encompass a wide range of AI topics such as knowledge systems, supervised machine learning (ML), and AI ethics. Our three curricula featured AI concepts and technologies that have recently become more accessible to the public such as generative adversarial networks (GANs), affective perception, and autonomous robotics. In *Creative AI*, we have students learn about various forms of AI-generated media and encourage them to consider the societal implications of GANs such as the creation of deepfakes, and how misinformation can spread through social networks that students use (Ali et al., 2021a; DiPaola et al., 2021). In *Dancing with AI*, we introduced a suite of AI-powered block-based coding tools (Jordan et al., 2021) and learning modules in which students design, build, and reflect on interactive, movement-based, multimedia experiences. Finally, in *How to Train Your Robot*, we integrated a low-cost Bluetooth robot into machine learning lessons to allow students to build AI projects motivated by doing social good, such as helping someone in their community (Williams et al., 2021).

Our curricula are designed to incorporate active learning through hands-on activities, projects, embed ethics and critical reflection about societal implications into all lessons, and to lower barriers like access to resources for students and teachers. We focused on constructionist, project-based-learning and designed our workshops to fit into the school day. However, due to the restrictions caused by the COVID-19 health emergency, we translated our curricula from an in-person classroom format to a synchronous, online workshop. In the summer of 2020, we trained 11 middle school teachers (primarily from Title 1 schools) to co-teach workshops that served 78 students from 8 states across the USA. In this paper, we describe the theoretical grounding, design, deployment, learning gains, and curriculum assessment that we employed during our summer virtual workshops. We used a mixed methods approach to evaluate our three curricula and associated webtools, materials, and practices. Our work was guided by the following research questions:

1. What knowledge and perceptions of AI do middle school students have before they begin our workshops?
2. What kinds of understanding about AI do students demonstrate after engaging in our lessons, interactive activities, and tools? What student-driven projects did our curricula enable?

We conclude with recommendations for future AI curricula that seek to broaden access to K-12 AI education.

## Background

### K-12 AI Literacy

As applications of artificial intelligence become more common in the lives of children, it has become increasingly important to educate students about how AI technologies work and how they impact society. It is projected that by 2025, about half of all work tasks will be completed by automated systems (Leopold et al., 2018; U.S. Bureau of Labor Statistics, 2019). Students must be equipped with the skills to build and work with AI to meet the needs of a shifting workforce. This means not only teaching students about what AI is, but also enabling them to create with it, think critically about its impacts, and advocate for responsible use (Ali et al., 2019; Zimmerman, 2018).

In recent years, researchers, educators, and technologists have come together to define what it means for K-12 students to be AI literate, drawing on inspiration from AI education at the collegiate level as well as K-12 computer science education (Kandlhofer et al., 2019; Lao, 2020; Long & Magerko, 2020; Touretzky et al., 2019b; Zimmerman, 2018; De La Higuera, 2019). The AI4K12 initiative, a collaborative effort between the Computer Science Teachers Association (CSTA) and Association for the Advancement of Artificial Intelligence (AAAI), was formalized in 2018 and defined the “Five Big Ideas of AI” that K-12 students must know. These are Perception, Representation and Reasoning, Learning, Natural Interaction, and Societal Impact. As academic interest in AI literacy for K-12 has increased globally, so has the number of available resources for K-12 AI education. For instance, the AI4K12 effort has created a website for educators, developers, and the general public to access the growing number of resources (www.ai4k12.org). Similarly, the MIT RAISE website (raise.mit.edu) highlights a wide range of K-12 AI literacy resources and curricula developed at MIT and the larger K-12 AI education community. Even the International Society for Technology in Education (ISTE) dedicated a section of its website^1^ to classroom AI resources, notably its hands-on AI activity guides that are available in different languages.

### Strategies for K-12 AI Education

Hands-on activities including unplugged simulations, programming activities, and tangible learning tools are prominent in many K-12 AI education resources. Unplugged activities have been used in computer science curricula to make computational ideas accessible without computers (Bell et al., 2009). For AI education, unplugged activities are powerful ways to have students explore the details of algorithms even if they do not have the background knowledge or the resources to actually program the algorithm (Ali et al., 2021a; Lee et al., 2021; Payne, 2020; TechGirlz, 2018). The “Human Neural Network” activity (TechGirlz, 2018) and “Contour to Classification Game” (Lee et al., 2021) delve into the finer details of machine learning algorithms without getting into the calculus that makes the algorithms work. These two papers are exemplary of how unplugged activities can make complex ideas in AI more digestible for younger learners.

Existing curricula and workshops often leverage novice-friendly coding tools to give students the opportunity to become designers of their own AI systems.

Tools like the *Personal Audio Classifier* and *Personal Image Classifier* from MIT App Inventor (Tang, 2019), Google’s Teachable Machine (Carney et al., 2020), and PlushPal (Tseng et al., 2021) allow students and educators to train, test, and export machine learning models using just their laptops and web browsers. Other tools, such as Google’s Slice of ML,^2^ Machine Learning for Kids (ML4Kids),^3^ and AlpacaML (Zimmermann-Niefield et al., 2019) allow students to build machine learning algorithms and get a glimpse into the black box. Several tools integrate machine learning model creation into block-based programming environments that many students are already familiar with. ML4Kids, Cognimates (Druga, 2018), PoseBlocks (Jordan et al., 2021), LearningML (García et al., 2020), and the Scratch Text Classifier (Reddy et al., 2021) are AI-integrated extensions for the open source, block-based programming language Scratch (Resnick et al., 2009). Similarly, AI Programming for eCraft2Learn^4^ is an extension of the drag-and-drop programming language Snap! (Harvey et al., 2013). Other tools utilize the generative aspects of AI such as GANs to allow students to creatively express themselves, such as GANimals (Boulais et al., 2020), GANPaint (Bau et al., 2019), MagicDraw (Ali et al., 2020), and pix2pix (Isola et al., 2017).

Finally, building on work in mathematics education research, physical manipulatives have been used in AI education to make ideas more accessible and concrete to students (National Council of Supervisors of Mathematics, 2013; Papert, 1980). Scratch Nodes (Hitron et al., 2018) and PlushPal (Tseng et al., 2021) use physical artifacts as a part of their machine learning courses to entice younger learners. At the university level, professors have seen the benefits of using robots in their AI courses to give physical meaning to the algorithms that they discuss in class (Kumar, 2004). However, robots may disrupt the learning process as they do not always work reliably and may consume precious class time while students assemble and debug their bots. Therefore, it is important that educators find a balance between learning about AI and assembling robot kits (Koski et al., 2008; Talaga & Oh, 2009). Similar benefits and challenges with using robots to teach AI exist at the K-12 level (Touretzky & Gardner-McCune, 2018; Williams et al., 2019).

### Opportunities for K-12 AI Education

Existing resources for K-12 AI education are generally short, informal learning opportunities that focus on a particular concept or big idea in AI. However, as De la Higuera (2019) argues, schools should make time to teach AI in their classrooms given the relevance of AI in children’s lives. This paper details our steps toward making curricula that are practical for classroom by centering student and teacher needs in our design. There are few published pieces on preparing in-service teachers to bring AI education to their classrooms. In one of the most notable examples, Vazhayil et al. (2019) trained 24 secondary school teachers (for students ages 14 to 18) to teach a project-based AI curriculum based on ML4Kids (Vazhayil et al., 2019). The research uncovered potential barriers for broader adoption of such a curriculum, including making tools that are suitable for classrooms with different technological needs, considering the pedagogical strategies used to deliver content, and ensuring that teachers had the knowledge and resources they needed to support their students as they learned. We designed our curricula to address these issues.

The ubiquity of machine learning algorithms makes them a relatable target for students growing up in this AI-infused age, hence most educational resources teach the basics of supervised machine learning (Marques et al., 2020). More recently, K-12 curricula developed by academic researchers are moving beyond machine learning and teaching more cutting-edge AI topics to students. For instance, generative ML, or AI that can generate new media, is a recent innovation in the field of AI. One of the most common algorithms used for generative ML is called generative adversarial networks, or GANs (Goodfellow, et al., 2014). While GANs can serve as a tool for enhancing creativity in children (Ali et al., 2020), they can also be used to generate fake media that is meant to deceive others (Nguyen et al., 2019). Generative AI tools have been used to create deepfake photos and videos that circulate on popular social media websites such as Twitter, Facebook, and Instagram. Students begin to be exposed to AI generated media in middle school, as they make their first social media accounts. Unfortunately, to our knowledge, there are few other published curricula on teaching K-12 students about generative ML. They are being taught in a handful of college level courses, such as Machine Learning for Artists,^5^ Computer Visions^6^; Artists and Machine Intelligence,^7^ and Creative Machine Learning for Design.^8^ However, college courses are not accessible to the general public because they require a baseline knowledge of programming and costly computing resources. One of our curricula, *Creative AI,* tackles the topic of generative ML because of its relevance to students yet absence in other K-12 resources.

Collegiate AI courses have historically overlooked ethics, separating it from other lessons or isolating it in a separate course altogether (Fiesler et al., 2020). That is beginning to change as researchers such as those associated with MIT’s Schwarzman College of Computing have created case studies for engineering students to explore ethics. At the K-12 level, ethics at the center of AI4K12’s big ideas in AI. The centrality of ethics is critical as AI systems have historically been biased against marginalized groups such as women, people of African, Asian, and Latin descent, Indigenous peoples, and low-income individuals and communities (Buolamwini & Gebru, 2018; Eubanks, 2018; Noble, 2018; O'Neil, 2016). Skirpan et al. (2018) found that when students learn ethics throughout a computer science course, they think more holistically about the implications of the technology that they are building than if ethics is taught at the end of a course or in a different course all together. At the K-12 level, few papers discuss ethics with students and those that do focus on the legal and social implications of deployed or hypothetical systems (Lassnig, 2018; Opel et al., 2019). The *Middle School AI* + *Ethics Curriculum* is an exception to this rule; it adapts the approach of collegiate courses that embed ethics in technical lessons to develop students’ ethical design skills (DiPaola et al., 2020; Payne, 2020). Doing so enabled middle school students to apply ethical decision making to their AI projects and was highly engaging for the students (DiPaola et al., 2020). We build on the work of the *Middle School AI* + *Ethics Curriculum* and prioritize embedding ethics in our curricula.

### Prior Literature on These Curricula

This paper builds upon prior works published about all three curricula. Ali et al. (2021a) and DiPaola et al. (2021) did deep dives into *Creative AI*’s “How GANs Work” and “Exploring GANs” activities, respectively.

Ali et al. (2021b) built on those papers to describe how students’ new understandings of generative ML supported their ability to make policy decisions. We reference results from these three papers to make our case for the design principles we used to create the *Creative AI* curriculum. Lee et al. (2021) described the *DAILy* curriculum, which contains some of the same activities as the *Creative AI* curriculum but used formative assessments to measure changes in students ‘ understanding of AI. Jordan et al. (2021) presented the technical implementation of the PoseBlocks platform that undergirds the *Dancing with AI* curriculum. That paper analyzed student and teacher feedback after using the tool while here we describe our design motivations for the tool and its accompanying activities. Similarly, Reddy et al. (2021) detailed the technical implementation of the text classifier we use in the *How to Train Your Robot* curriculum and Williams et al. (2021) analyzed teacher feedback on two iterations of *How to Train Your Robot*. Compared to those prior works, this paper offers further analysis of students’ performance on activities, the design of the curricula through the lens of design principles, and discussion on the three curricula in conversation with one another.

## Overview of Curricular Designs

### Design Principles

All three of our curricula incorporated three key design principles: 1) active learning, 2) embedded ethics, and 3) low barriers to access. We used these principles to inform the pedagogical strategies for the learning objectives, activities, assessments, and tools of each curriculum as applied to their respective AI topics.

#### Active Learning

Active Learning, the first key design principle, is an instructional method in which students play a key role in their learning by engaging in activities then processing information through reflection (Bonwell & Eison, 1991; Michael & Modell, 2003). Rather than passively engaging with material through teacher lectures, students drive the learning process. Research shows that active learning leads to higher information retention, more profound absorption of ideas, and more positive attitudes toward the subject—especially in the sciences (Michael, 2006; Prince, 2005). Furthermore, active learning builds on prior knowledge making it an appropriate instructional strategy for students who are new to a field of study (McConnell, 1996; Michael & Modell, 2003). Most middle school students lack the mathematical and computational background to understand AI as it is traditionally taught in undergraduate classrooms. With active learning, students can synthesize an evidence-based understanding of algorithms by personally encountering new ideas through construction and discovery (Bruner, 1961; Fortus, 2004; Papert, 1980).

In our virtual workshops, students engaged in active learning through hands-on activities, often discovery activities done in small groups, and then refined their understanding with questions and group discussion (Bruner, 1961; Michael & Modell, 2003). Activities included demos of existing AI systems, simulations that allowed students to function as different components of an algorithm, and group discussions about how humans and computers accomplish cognitive tasks. We also engaged students in active learning by designing and constructing artifacts (Fortus, 2004; Kafai, 1995; Kolodner et al., 2003; Papert, 1980). All three curricula ended in a final project and presentation, which enabled students to apply what they learned to a personally meaningful project that they could critique, reflect upon, and share with others. Examples of active learning activities from each curriculum are featured in the first row of Table 1.

**Table 1:** Examples of how the design principles (active learning, embedded ethics, and low barriers to access) were applied to our *Creative AI*, *Dancing with AI*, and *How to Train your Robot* curricula

	*Creative AI*	*Dancing with AI*	*How to Train your Robot*
Active Learning	In the “How GANs Work” activity, students embodied the roles of a generator and a discriminator in a game about the relationship between the two neural networks	In the “Charades” activity, students learned about feature selection. In a game, they practiced identifying, describing, and physically acting out salient features of movements to design movement classifiers	In the introduction to “Image Classification” activity, students experimented with the Quick Draw game and dataset to see how different features of images contribute to how algorithms learn to distinguish classes
Embedded Ethics	As part of the “Exploring GANs” activity, students play with various GAN demos then discuss potentialt positive and negative impactsof the tools	As students learn about how image classification works in the “Examples of Classification in AI” activity, they reflect on real world implications of algorithms trained with flaws in their datasets	All students completed and presented ethical matrices for their final projects. In the matrices, students identified the key stakeholders and potential good and bad consequences of deploying their projects
Low Barriers to Access	GANs typically require a lot of computing power to create The “GAN Play” and “Text Generation” tools used pre-trained models to allow students to run models on a web browser and quickly create text and image artifacts	PoseBlocks are designed to be web-first and to do real-time affective and image recognition in a web browser. Additional care was taken to ensure that students could use PoseBlocks even if they did not have a dedicated GPU, camera, or microphone on their devices	There is no programming pre-requisite for the curriculum. The first programming activity uses a set of mini challenges to get students up to speed on the important robotics and programming concepts they will need later in the class

#### Embedded Ethics

The second principle, embedded ethics, refers to the pedagogical practice of teaching technical and ethical concepts in tandem (Payne, 2020; Saltz et al., 2019). Ethics is a key learning objective included in K-12 AI frameworks as Big Idea #5 in Touretzky et al. (2019a, b) and Competency #16 in Long and Magerko (2020).

The benefits of embedding ethics into technical lessons include students developing a better understanding of how technology interacts with society and increased engagement (Payne, 2020; Saltz et al., 2019). Practices for embedding ethics include using real world examples to contextualize lessons, critiquing AI systems, and using stakeholder analysis to inform system design (Payne, 2020; Register & Ko, 2020; Saltz et al., 2019; Shen et al., 2021). Where some middle school subjects may struggle to demonstrate the relevance of the material to students’ lives, AI ethics confronts students with developing, real-world issues that impact their lives every day. Furthermore, inviting students to bring their ideas into the classroom, and to hear about the perspectives and experiences of others, helps them develop human skills like empathy and critical thinking (Payne, 2020).

Each of our curricula focused on two key ideas in technology ethics: 1) viewing technology as a sociotechnical system (Winner, 1980) and 2) critiquing the ethical implications of specific technologies (i.e., GANs, facial recognition). We taught ethical concepts through experimentation, discussion, and real-world examples. For example, in the “Exploring Word Analogies” activity in the *How to Train Your* Robot curriculum, students use a visualization tool to explore gender, age, class, and other biases that exist in word embeddings. Many of the concepts that students engaged with are ongoing discussions in the field of AI. The irresoluteness of these topics encouraged students to embrace ambiguity and recognize the importance of making their voices heard. Discussions between students encouraged perspective taking and allowed students to debate points made by their peers. Additional instances of activities which emphasized embedded ethics are shown in the second row of Table 1.

#### Low Barriers to Access

Our third design principle, low barriers to access, involves centering student and teacher needs in our design as we strive toward the larger goal of reaching all students with AI education. To address the barrier of engagement, we incorporated AI with subjects like art, dancing, and robotics (e.g., the *Dancing with AI* curriculum used dancing as an opportunity to engage students in physical movement and embodied learning as they explored ideas in AI). We took this approach to appeal to students’ existing interests (Design Consideration #12 from Long & Magerko, 2020) and to make AI more approachable (Zimmermann-Niefield et al., 2019).

**Fig. 1:**
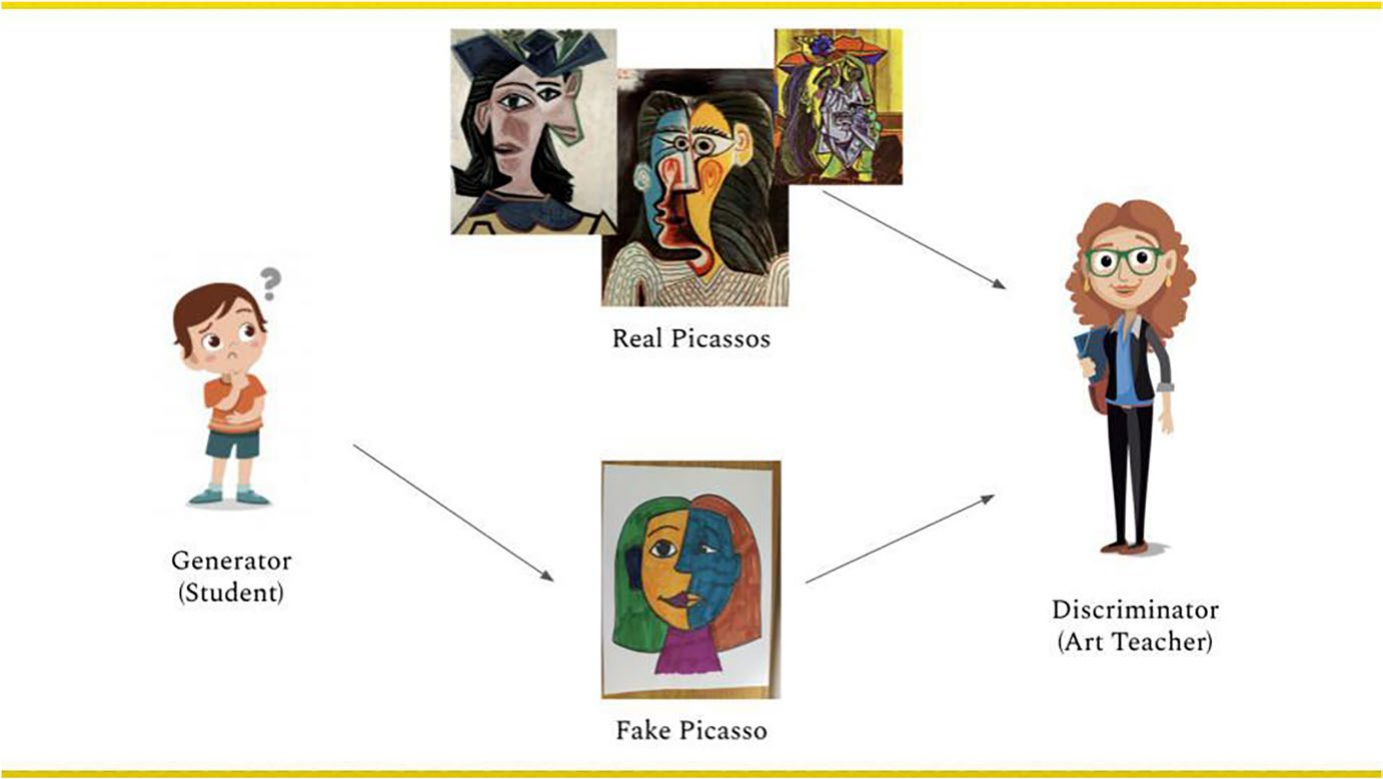
Visual representation of the student/art teacher analogy to describe the generator and discriminator

To reduce the complexity of AI concepts, we decomposed concepts into their key ideas (Design Consideration #5 from Long & Magerko, 2020) and leveraged unplugged activities to teach students those ideas (Bell et al., 2009). The primary benefits of unplugged lessons are that they remove programming as a barrier to entry into computing ideas and rebuff the misconception that computer science is primarily about programming (Bell et al., 2009). Unplugged activities like role-playing, simulation, and physical manipulatives in AI curricula have made it possible for educators to present complex concepts without overwhelming students (Ali et al., 2021a; DiPaola et al., 2020, 2021; Payne, 2020; TechGirlz, 2018). An example from the *Creative AI* curriculum is an analogy about a student and an art teacher to present the roles of generator networks and discriminator networks in general-adversarial networks (GANs) (Fig. 1). The analogy was relevant to students’ personal experiences creating art in an art class, enabling them to comprehend an abstract AI concept that might have otherwise been difficult to grasp.

Once students have a foundational understanding of AI, programming activities can deepen their understanding through hands-on practice. However, the need for extensive computing resources can make it infeasible to bring hands-on AI lessons into the classroom.

**Fig. 2:**
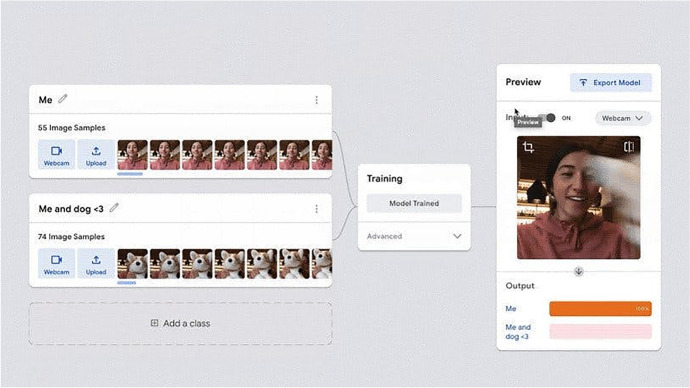
Screenshot of the Teachable Machine interface, which abstracts away technical implementation details, and advanced mathematics from student users. (Image from teachablemachine.withgoogle.com website.)

Online tools such as the Teachable Machine (Fig. 2), Cognimates, Machine Learning for Kids,^9^ LearningML, and our AI Blocks have made it easier for students to train AI models without needing expensive, high-end hardware (Carney et al., 2020; Druga, 2018; García et al., 2020; Jordan et al., 2021). *Dancing with AI* and *How to Train Your Robot* heavily leveraged block-based programming languages, which support beginners by abstracting away superfluous technical details, to give students hands-on, AI creation opportunities. More examples of how we addressed barriers to access in each curriculum is available in the third row of Table 1.

### Curricula Descriptions

We applied the aforementioned curriculum design principles to three AI education curricula – *Creative AI*, *Dancing with AI*, and *How to Train your Robot*. A comprehensive list of curricula activities can be found in Appendixes 1, 2 and 3. More information about these curricula can also be accessed at https://raise.mit.edu. Each curriculum not only addresses the shortage of AI curricula for non-experts, but they do so in a manner that allows students to understand the technology’s relevance to their own lives.

We designed our three curricula to promote the following learning outcomes vis the specific content and active learning experiences for each topic:

- **Technical AI Knowledge:** Students can define AI and can identify AI systems in their daily lives and articulate what makes them AI. Students have a practical understanding of how AI algorithms (pertaining to each curriculum) function and humans’ role in creating AI systems.
- **Ability to Think Critically about the Implications of AI:** Students can think critically about the potential benefits or harms of AI systems and their impact on stakeholders. Students reflect on and discuss ethical issues (e.g., fairness, privacy, and responsible design) as they relate to AI.
- **Ability to Apply AI Knowledge:** Students will be able to appropriately employ their AI knowledge and skills to topics they personally care about. Students can leverage skills like ethical thinking, creativity, empathy, and idea synthesis as they create AI artifacts.

#### Creative AI

Despite the widespread use and misuse of generative ML in the media, there are few existing efforts that educate school-age children on how AI can generate media and the implications of this technology. The goal of this curriculum is to teach middle-school children about creative ML techniques and how people can partner with AI to create digital art and expressive media. Students explored tools and techniques such as neural networks and generative adversarial networks (GANs) across various forms of media including text, images, and music. We developed web-based tools that allow students to input their own text and drawings to generate new media, for instance to create stories and illustrations using GANs. Throughout the workshop, we discussed important ethical issues surrounding generative AI art that allowed students to reflect on how machine-created art differs from human-created art. Finally, students learned about deepfakes, or fake media created by generative models that can be used to deceive others. Students attempted to identify deepfakes, discussed the harms of fake media, and learned about how misinformation, like deepfakes, can spread online. Specific information about the activities in this curriculum can be found in Appendix 1.

#### Dancing with AI

Many middle school students have interests in dance, art, physical movement in sports, and video games with gestural interfaces. However, it is difficult to engage with these interests in existing block-based coding environments. In this workshop, we introduce a suite of AI-powered block-based coding tools and learning modules in which students design, build, and reflect on interactive, movement-based, multimedia experiences through a user-friendly gestural interface. Students built projects with our two new sets of AI-powered blocks: hand/body/face position-tracking and expression-detecting blocks, and blocks that allow students to import their own image- and pose-recognition models trained with Google’s Teachable Machine. Our programming platform^10^ leverages TensorflowJS (Smilkov et al., 2019), MediaPipe (Zhang et al., 2020), and the Scratch Blocks open-source code repository^11^ to provide a browser-friendly programming platform (Jordan et al., 2021).

Students design and build AI-powered projects that align with their interests, train their own supervised machine learning models, and reason about the ethics and presence of AI systems in their everyday lives. Specific information about the activities in this curriculum can be found in Appendix 2.

#### How to Train Your Robot

In the *How to Train Your Robot* curriculum, students learn about AI technologies relevant to machine learning and autonomous robotics, including speech and image recognition (Williams et al., 2021). They learn about supervised machine learning, how to train models for use in applications and robotic agents, ways that ML models can be vulnerable to error, and ethical design principles.

We developed a custom AI Blocks programming platform,^12^ forked from the open-source Scratch Blocks code repository.^13^ With these AI blocks, students can integrate image recognition models from Google’s Teachable Machine, text classification models, and the robot with everything else that Scratch has to offer. Students used our robot platform, AI blocks, and ethical design methodologies to build AI projects that serve and/or entertain others.

This curriculum and its associated robot kit was originally designed for and deployed in classrooms for 30 h of in-person lessons. In the online version of the workshop, we mailed Bluetooth robots^14^ (currently priced at $40 each) and allowed students to keep the robots. The robot component proved effective in increasing students’ excitement about the topic and aided their understanding of computational ideas. Specific information about the activities in this curriculum can be found in Appendix 3.

## Methodology

### Research Questions

We collaborated with middle school teachers to pilot our curricula. To assess all three curricula, we used a mixed-methods approach that was guided by the following research questions:

1. What knowledge and perceptions of AI do middle school students have before they begin our workshop?
2. What kinds of understanding about AI do students demonstrate after engaging in our lessons, interactive activities, and tools? What student-driven projects did our curricula enable?

### Online Study Context

Due to the health emergency caused by an outbreak of the COVID-19 virus in 2020, we were faced with the challenge of teaching our three new project-based curricula, modified from in-person formats, using remote online learning techniques. All three curricula were deployed in synchronous online summer workshops, where students used Zoom video conferencing on their personal devices to participate, often a Chromebook, but students also used iPads and other mobile devices. Workshops were run over the course of a single week (Monday to Friday) with daily sessions that spanned 2–2.5 h (time-reduced from the in-person versions to prevent screen fatigue).

It was challenging to maintain the fidelity of the tangible and discussion-heavy activities in a virtual setting since some students did not have technical resources (microphone, camera, stable Internet connection), were uncomfortable speaking out loud, or found it hard to stay engaged over a video call. We adapted our curricula and teaching styles to accommodate student needs by using new and familiar collaborative classroom tools (namely Google Classroom, Google Slides, and Google Forms) for students’ activities. To encourage collaboration, we made use of synchronous web tools, such as Google Sheets, and interactive tools developed using web-sockets so that they could see each other’s work in real-time. This also allowed teachers to view student work and help debug in real-time. Students used breakout rooms and chat-based interactions to collaborate with one another, both to share their ideas and ask for help. To aid in code debugging, we provided students with instructions in different modalities, such as illustrated guides and video tutorials.

### Teacher Training and Workshop Role

All three workshops were taught in partnership with middle-school teachers that we recruited by leveraging Amazon Future Engineer mailing list. From a roster of 59 teachers who expressed interest in the program, we recruited 11 teachers from around the United States (California, Florida, Georgia, New Jersey, New York, Ohio, and Texas). We recruited two to five middle school teachers for each workshop based on their teaching background and expressed reason for interest. We compensated them $500 for their participation.

Teachers were simultaneously recruiters, trainees participating in professional development, instructors, and teaching assistants. The professional development for running the course took place before the workshops and at the end of every day the workshop was offered. In these sessions, we reflected on the previous days lessons and ran through the activities for the following days. We encouraged teachers to actively shape the workshops by modifying existing lessons and introducing new ideas. Since each workshop took place twice, we were able to implement feedback from teachers in the second round. After the workshops were completed, we expected that teachers would become ambassadors for AI education, bringing what they had learned into their classrooms and schools.

### Participant Demographics

The Creative AI, Dancing with AI, and How to Train Your Robot curricula were taught as three separate virtual workshops, each offered twice. The study protocol was reviewed and approved by the Institutional Review Board at Massachusetts Institute of Technology. Parents and students signed consent and assent forms, respectively, to participate in the study. All the students were informed that the workshop was a part of a research study, and that information would be collected to evaluate and refine each workshop and associated materials. Further, participants were assured that they could withdraw from the study at any point.

The authors, the majority of whom are women, African American, Asian American, and/or from low-income backgrounds prioritized inclusivity in recruiting and conducting this study. We partnered with teachers to recruit students from a wide range of backgrounds with varying levels of interest and exposure to Computer Science. Teachers recruited between 6—12 middle school students, depending on the capacity of the workshop, and strove for a 50/50 gender balance. A total of 78 students participated in the study ranging in age from 12.44 ± 1.32 years old (Table 2). Six (6) students participated in two workshops.

**Table 2:** Students demographic information for each workshop

Workshop	Number of students	Age	Gender
*Creative AI*	38	12.605 ± 0.975	F = 18, M = 20
*Dancing with AI*	21	11.857 ± 1.283	F = 12, M = 9
*How to Train Your Robot*	25	12.696 ± 1.343	F = 16, M = 6, Prefer not to say = 3

We do not report students’ race or socioeconomic background because we do not analyze our results through the lens of these sensitive demographic factors. However, we asked teachers to recruit students that were representative of the populations of their schools.

Six (6) out of 10 participating schools were Title 1 schools. The United States of America designates schools as Title 1 if at least 40% of students come from households below the poverty threshold.^15^ Of the remaining schools, one was a private, charter school where 96% of the students are African, Hispanic, or Indigenous Americans. Another school was a homeschool organization for students with special needs and their families; all their students are African Americans. The third school was a public, magnet school that serves students in rural midwestern communities. And the fourth school was a public school where 32.8% of students are African, Hispanic, or Indigenous Americans.

### Data Collection

#### Pre-Questionnaire

To assess participants’ familiarity with and perceptions of Artificial Intelligence, students were administered a pre-test questionnaire using a Google Form on the first day of the study. The questions spanned across three categories: prior experience, or their existing familiarity with AI and technology; AI perception, or their current understanding of the capabilities of AI; and self-perception, or their understanding of themselves and their relationships with AI. A full list of questions can be found in Appendices D through F.

#### Workshop-Specific Assessments

The remainder of the assessments occurred in the context of the activities that students completed in each workshop. These assessments were tailored to the content and activities that students did in each workshop. We analyzed them using mixed quantitative and qualitative approaches to capture rich information about how much students learned. Specifically, we used:

- Statistical metrics to compare pre-post questionnaires about students’ understanding of AI concepts.
- Thematic coding based on grounded coding theory (Thornberg and Charmaz, 2014) to inspect the conclusions (and misconceptions) students had about AI after completing activities.
- Rubrics to evaluate the projects students generated as they applied their understanding of technical and ethical concepts to problems of personal interest.

## Results

In this section, we seek to answer our two research questions:

1. What knowledge and perceptions of AI do middle school students have before they begin our workshop?
2. What kinds of understanding about AI do students demonstrate after engaging in our lessons, interactive activities, and tools? What open-ended projects did students make with our AI tools?

We will explore these questions by analyzing students’ response to the pre-test and post-test questionnaires as well as their engagement and performance in the lessons and activities.

### Students’ Understanding and Perceptions of AI across all Workshops

#### Prior Experience and Familiarity with AI

**Fig. 3:**
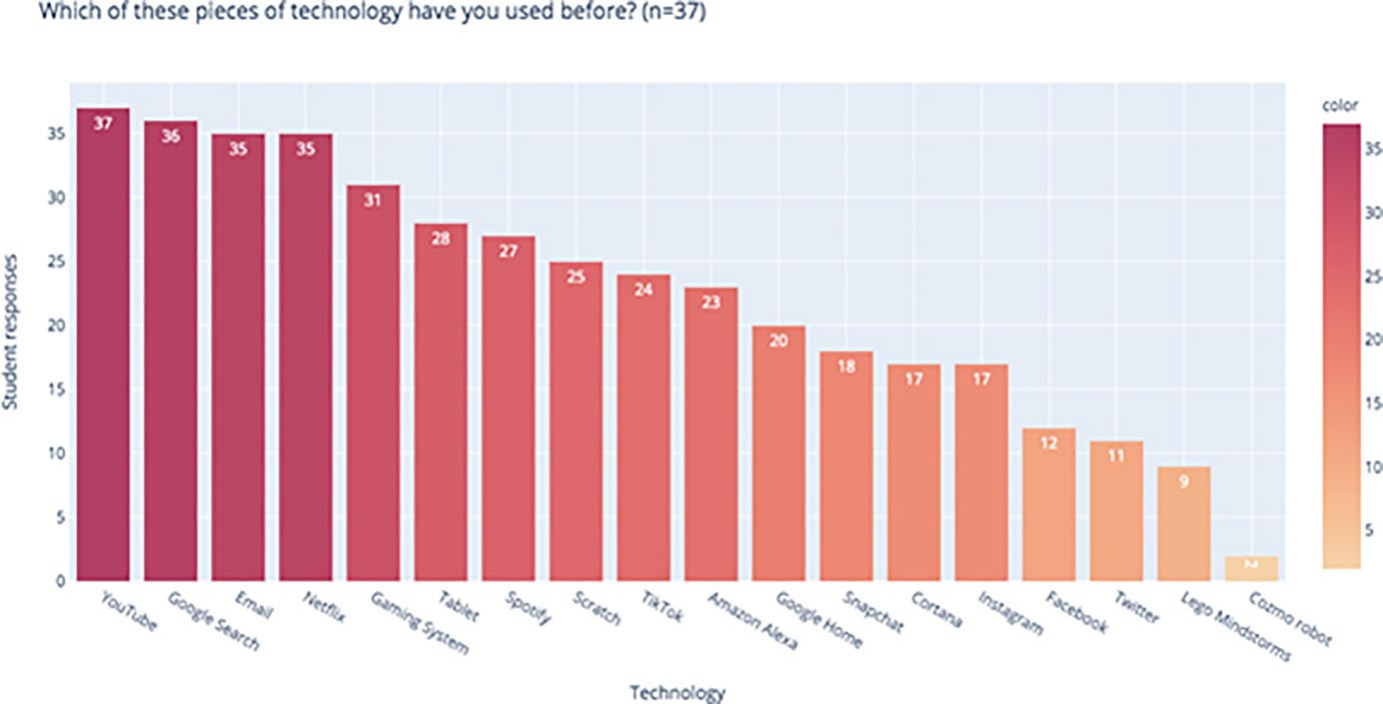
Most students across the three workshops were also active users of technologies that leverage AI, especially YouTube, Google Search, Email, Netflix, Tablets and Gaming Systems

We found that students were overall extremely aware of the existence of artificial intelligence. Across all three curricula, 90.5% (*n* = 74 total responses) of students had heard of AI before and an overwhelming majority of students had interacted with AI integrated technologies such as YouTube (100%, *n* = 45), Google Search (95.6%, *n* = 43), and Netflix (88.9%, *n* = 40) before (Fig. 3). When we asked students to define AI, the most common words in their definitions were “intelligence” (54.1%, *n* = 37), “human(s)” (48.6%, *n* = 37), “artificial” (37.8%, *n* = 37), “machines” (32.4%, *n* = 37), “robots” (29.7, *n* = 37), “computer” (29.7%, *n* = 37), and “learn” (16.2%, *n* = 37).

Related to students’ prior experiences with technology, we also wanted to understand the extent to which they recognized how much they used AI in everyday life. We asked if the following technologies used AI: Google Search, Wireless Printers, Video Calls (e.g., Facetime, Zoom), Game Consoles (e.g. Xbox, Switch), YouTube (“Up next” recommendations), Movie Recommendations (Netflix, Amazon Prime, Hulu), Snapchat Filters, GPS Apps (e.g. Google Maps, Waze), and Voice Assistants (e.g. Alexa, Siri, Google Home). Except for Video Calls, more than half of students said that *every* example used AI.

Students were most confident that Voice Assistants (93.2% “Does use AI”, *n* = 44), Google Search (79.6%, *n* = 44), Game Consoles (77.3%, *n* = 44), and GPS Apps (72.7%, *n* = 44) use AI. Since students erred on the side of calling items AI, the majority correctly identified the examples that truly were AI: Google Search, YouTube’s “Up next” recommendations, Movie Recommendations, Snapchat filters, GPS Apps, and Voice Assistants. Students were least sure whether Video Calls (21.6% said “Not sure this uses AI”, *n* = 37), Game Consoles (13.64%, *n* = 44), Snapchat Filters (13.62%, *n* = 44), and Wireless Printers (13.51%, *n* = 37) used AI. These results point to the need to help students systematically reason through what truly makes something AI.

#### Students’ Perceptions of AI

**Fig. 4:**
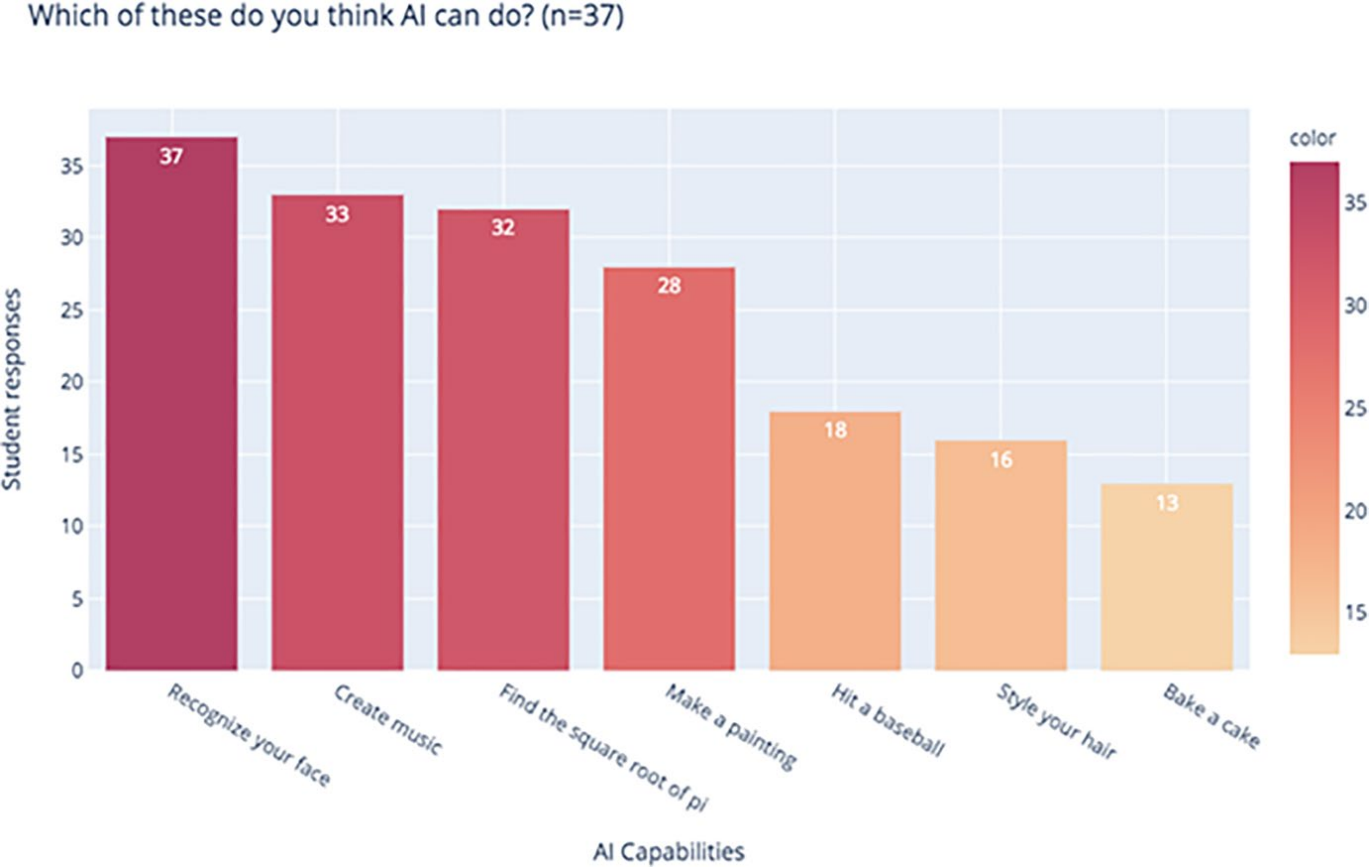
Students’ perceptions of capabilities of AI

When asked about what they think AI can do, applications such as mathematical operations and face recognition were most popular. Students also believed that AI could create music (89.2%, *n* = 37) and make a painting (77.8%, *n* = 37). Responses such as baking a cake (35.1%, *n* = 37), styling hair (43.2%, *n* = 37) and hitting a baseball (48.6%, *n* = 37) were less popular. Media portrayal of AI capabilities, as well as AI tools in applications familiar to children (such as face recognition in social media) seemed to have an influence in their perception of AI capabilities (Fig. 4).

**Fig. 5:**
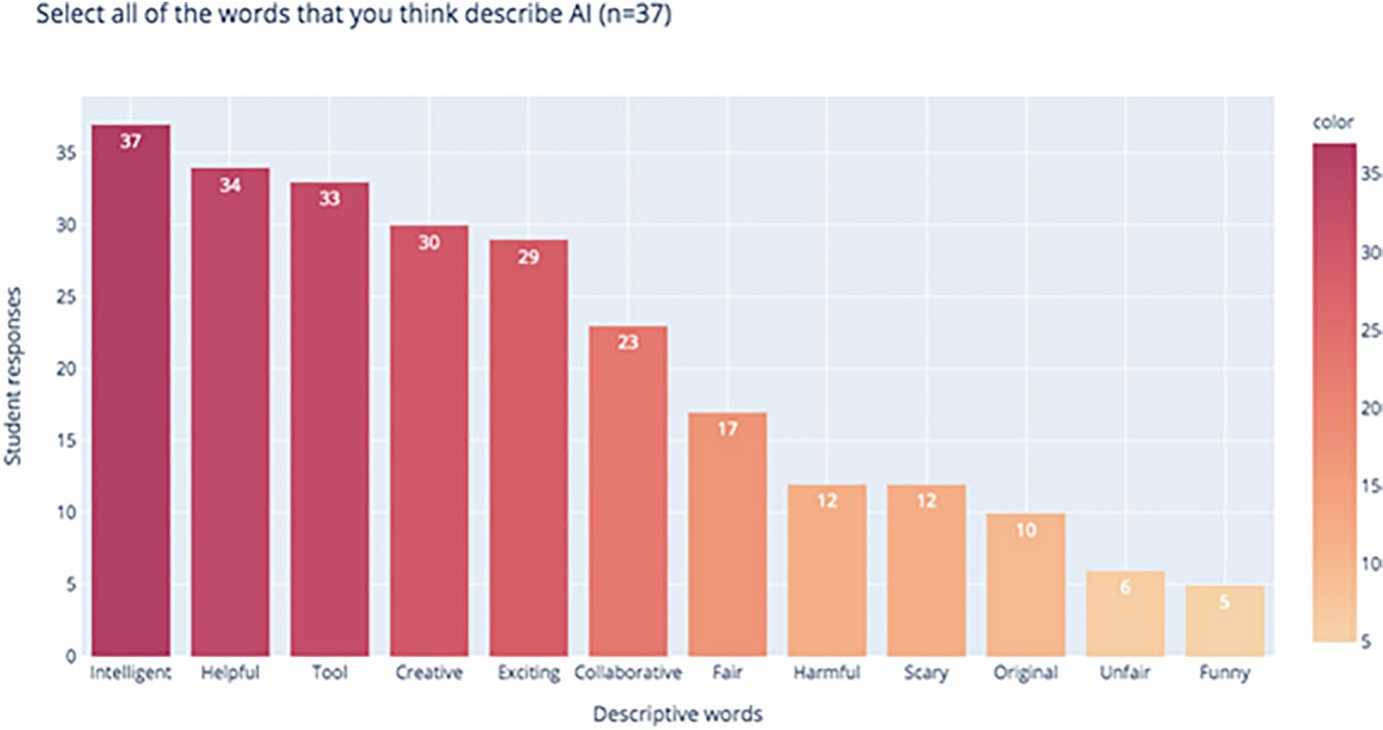
Terms that students used to describe AI

In terms of how they saw AI playing out in technology, more students believed that AI would make jobs easier (73.0%, *n* = 37) rather than take over jobs. These students represented one group of respondents who had a positive view of AI. The 10 students who answered that AI can take over jobs also described AI as scary and potentially harmful (Fig. 5).

#### Students’ Attitudes toward Learning AI

**Fig. 6:**
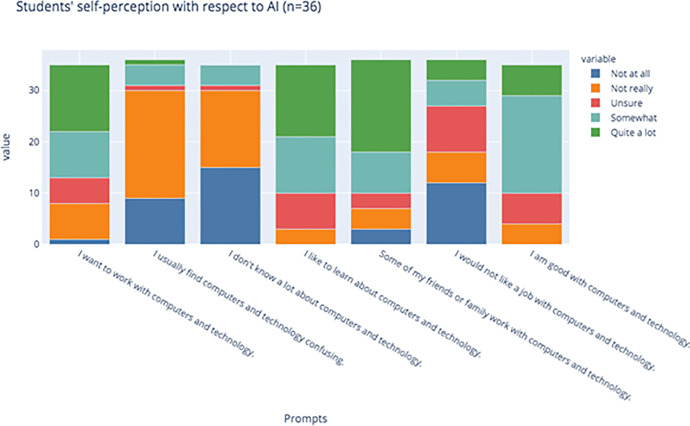
Students’ self-perception in relation to AI technologies

We observed that only a small minority of students answered that they were smarter than AI (17.1%, *n* = 35), and only 33.3% (n = 37) of students believed that they exclusively could exclusively control AI. Students seemed to believe in the collaborative potential of AI, however, with 81.1% (*n* = 37) reporting that they believed the relationship between themselves and AI was symbiotic, such that humans and AI could both have control of technology (Fig. 6).

In the pre-test, students also showed that 62.9% (*n* = 35 total responses) of students want to work with computers and technology in the future, and 71.4% considered themselves to be tech savvy; only 14.3% of students reported that they found computers confusing, and only 11.4% (*n* = 35) said that they do not know a lot about computers. Unsurprisingly, 71.4% (*n* = 35) of students also reported being interested in learning about technology; students in these pilots were hand-picked by their teachers as good candidates for participating in the classes. Out of (*n* = 35) students, 74.3% students had family members or friends who worked with computers and technology.

Throughout their workshops, students completed activities that we used to collect data about their understanding of AI concepts. The next section reports the results of students’ technical and ethical understanding of lessons from the *Creative AI, Dancing with AI,* and *How to Train Your Robot* lessons.

### Students’ Understanding of Creative AI Lessons

The key objectives for the *Creative AI* curriculum are to have students understand GANs and how machines generate art. Students explored positive, like human-AI collaborative art, and negative, like fake media and disinformation, applications of generative algorithms that we see today.

The learning objectives of *Creative AI* include:

- **Understanding generative algorithms and their applications**: Students can describe how GANs work, infer how different examples of GANs were built, and apply techniques to determine if a piece of media was likely to be produced by GANs
- **Foreseeing the social impact of algorithms:** Students can evaluate a GAN system for the potential beneficial and harmful ways it may be used in society
- **Creating collaborative human-AI artifacts:** Students can use generative tools for creative expression

The following sections explore students’ performance on the activities related to these ideas. Students responded to these questions as a part of the workshop activities or during a reflection period at the end of the day. Students created art with generative algorithms on the last day of the workshop.

#### Technical Understanding: How GANs work

We asked students two assessment questions before and after the workshop. The first question had three parts where students had to mark statements about GANs as “True” or “False”. Their responses are shown in Table 3.

**Table 3:** Number of correct responses for each statement, n = 11 responses

Question Statement	Correct Answer	# Correct (pre-test, out of 11)	# Correct (post-test, out of 11)
Q1.1—A generator and discriminator are both neural networks	True	7	8
Q1.2—The generator and discriminator are working in competition with one another	True	2	4
Q1.3—The discriminator gives feedback to the generator	True	5	8

The second question asked students about how GANs work: “A GAN is being trained to generate images of clouds. The generator creates an image and sends it over to the discriminator. The discriminator does not classify the image as a cloud. What happens next?” Students had to choose the correct answer out of four options: a) the discriminator tries to generate a new image this time, b—correct) the generator generates a new image based on feedback from the discriminator, c) the discriminator changes the dataset it is trained on, d) the generator generates a new image randomly and sends it back to the discriminator, or e) I am unsure. A total of seventeen (17) students answered this question. More students selected the correct answer, b, at the end of the workshop (70.6%) versus the beginning of the workshop (41.2%).

**Fig. 7:**
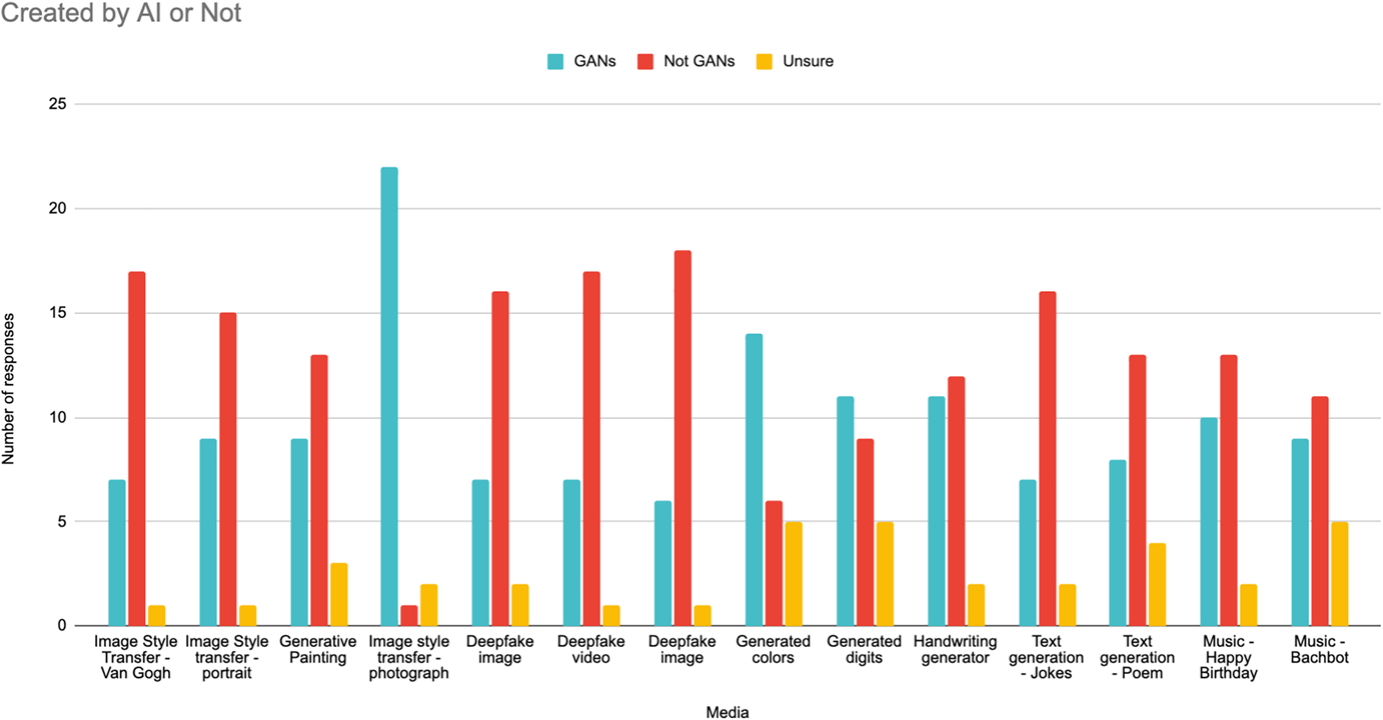
Students’ responses when they were asked if certain media were GAN-generated or not (they were all GAN-generated)

With the goal of understanding what students believe AI can create, in the GANs or Not activity, students judged whether fourteen distinct pieces of generative media were created by a generative model or not. Media consisted of photos, audio, and text, and was copied into Google Slides. The correct answer to all questions was “Yes, the media was made by a generative tool.” A full breakdown of student responses can be found in Fig. 7. More students thought that the Style Transfer image, generated colors, and generated digits were created by a GAN, relative to those who did not think it was created by a GAN.

**Fig. 8:**
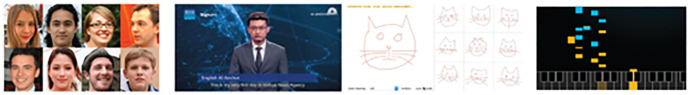
GAN Tools (left to right): 1) This Person Does Not Exist is a website that curates fake faces generated using StyleGan2 that has been trained on human faces to generate fake human faces using GANs (Karras et al., 2019). 2) Developed by Xinhua and the Chinese search engine, these AI-powered news anchors were developed through machine learning to simulate the voice, facial movements, and gestures of real-life broadcasters (Kuo, 2018). 3) Built by Google Creative Lab, Sketch RNN is an interactive web experiment that lets you draw together with a recurrent neural network model (Ha & Eck, 2017). 4) Built by Yotam Mann and Google, this web tool utilizes generative piano music to let users play a duet with the computer. Users press keys to play a music note, and AI Duet adds some notes to form a duet (Karras et al., 2019)

In the Exploring GANs activity, students explored up to four web tools that utilize generative ML. Students had 10 min to try out four different GAN tools that are hosted online (Fig. 8). After they explored these tools, they were asked to pick one and identify: 1) what the generator in the GAN was trying to generate, and 2) what dataset the discriminator in the GAN was basing its decisions on. Students could respond for up to four tools depending on how long it took. Of the 58 responses, 65% of student responses were able to correctly identify what the generator was trying to generate, and 61% of student responses were able to correctly identify the dataset that the discriminator was trained on.

#### Ethical Understanding: Understanding the Societal Implications of Generative Machine Learning

We asked students to consider the ethical implications of GANs by identifying the potential benefits and harms of the GAN tools they explored in the Exploring GANs activity. Two researchers grouped students’ responses into clusters, achieving a percent agreement of 92%. As an example, researchers coded a students’ response “teach kids how to draw” as a benefit of Sketch-RNN under “learning.”

**Fig. 9:**
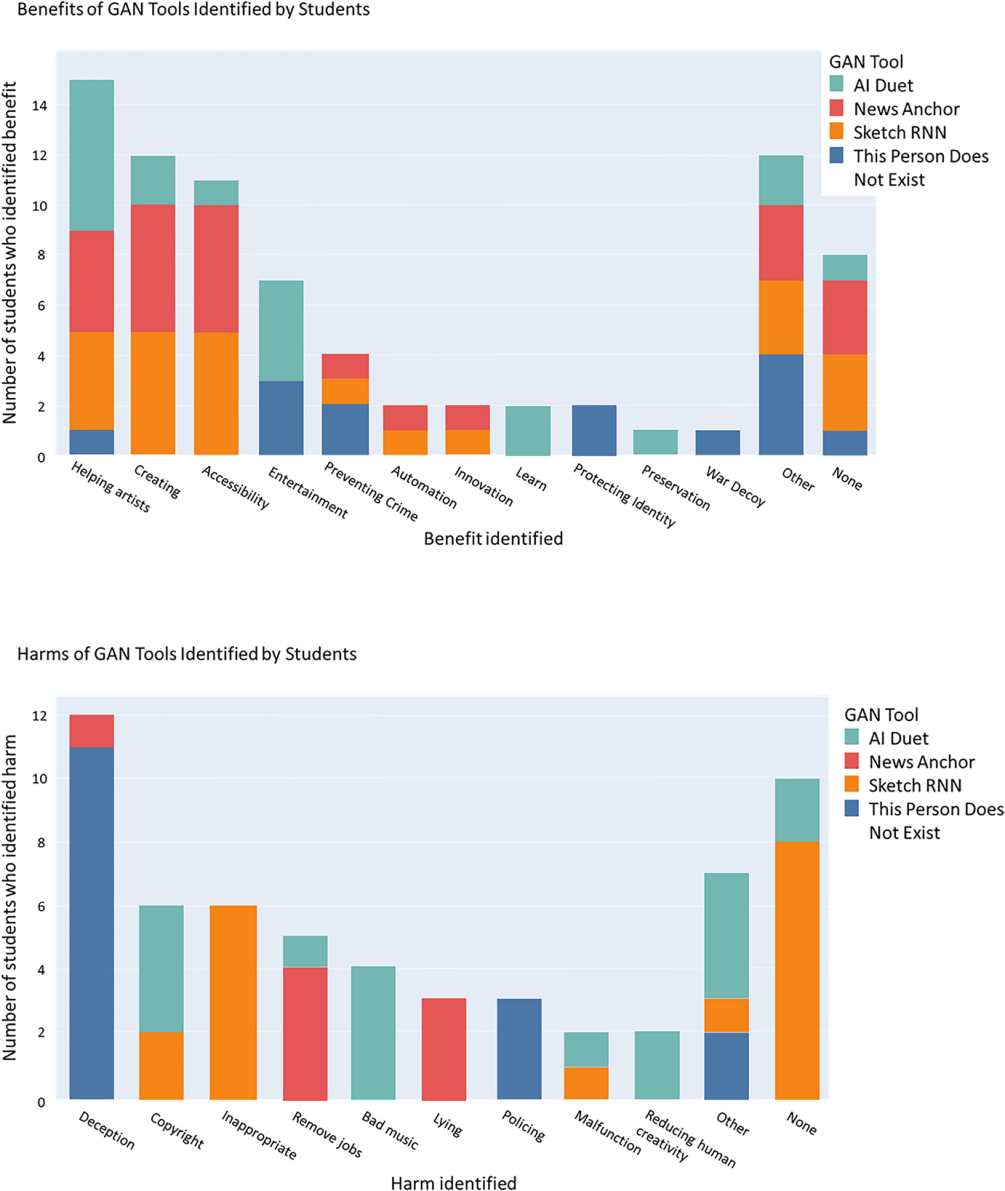
Students identified benefits (top) and harms (bottom) of the GAN tools that they explored

The 58 students who submitted responses reported a more potential benefits (79) than harms (60) as shown in Fig. 9. There was a difference in the number of benefits and harms identified for the kinds of tools. We observed that students identified more benefits of purely artistic tools such as Sketch RNN and AI Duet and more harms of GAN tools that generated human faces or videos such as AI News anchor or This Person Does Not Exist. Students associated these tools, which generate anthropomorphic media, with potential harms including “deception” or “policing.”

In the “Spotting Deepfakes” activity, students took to a quiz to identify whether something was a deepfake or not, learned strategies for spotting deepfakes, and then retook the same deepfake quiz. There was no significant change between the pre-quiz (M = 53.44, sd = 14.73) and post-quiz (M = 53.55, sd = 15.18), t(30) = -0.03, *p* = *0.98.*

Many students mentioned how difficult it was to tell if something was a deepfake or not, even after they had learned techniques to detect them:

“Today, the main thing I learned is how to see if a video or picture is a deepfake/made by AI. I also learned how AI can generate things like pictures and stories using data... I found the deepfake exercise really hard, this is because AI videos can be very convincing.” (Coral,^16^ female, 12 years old)

Students explained that difficulties came from the fact that they were required to “pay attention” to “small details”:

“The activity if we had to identify if it was a deepfake or not was hard. It was very hard to tell if it was. There are so many miniature details that you have to look at, and that makes it really tricky.” (Camila, female, 10 years old)

#### Applying Knowledge: Creating with GANs

A total of 33 students completed and submitted the final project – a story created with the text and image generator tools. The text generator tool allowed students to generate a story by applying a text style from one of 34 books or authors onto user-provided seed text. The image generator tool allowed them to create a drawing and then stylize it with a chosen visual style (e.g., a ‘cat’ style). Students tended to pick text styles that were more familiar or child friendly. For instance, 14 (42.42%, n = 33) students chose the Dr. Seuss style, 7 (21.21%, n = 33) chose the Harry Potter style, 3 (9.09%, n = 33) chose the Wizard of Oz style, 2 (6.06%, n = 33) chose Williams Shakespeare, Carl Sagan, Knock-knock jokes, and Life of Pi styles each. One student used the Dracula style. No students chose the more unfamiliar subjects such as Virginia Woolf or the novel *Pride or Prejudice*. The seed text that students provided the style generators were all based on personal or fictional narratives inspired by the generative images.

**Fig. 10:**
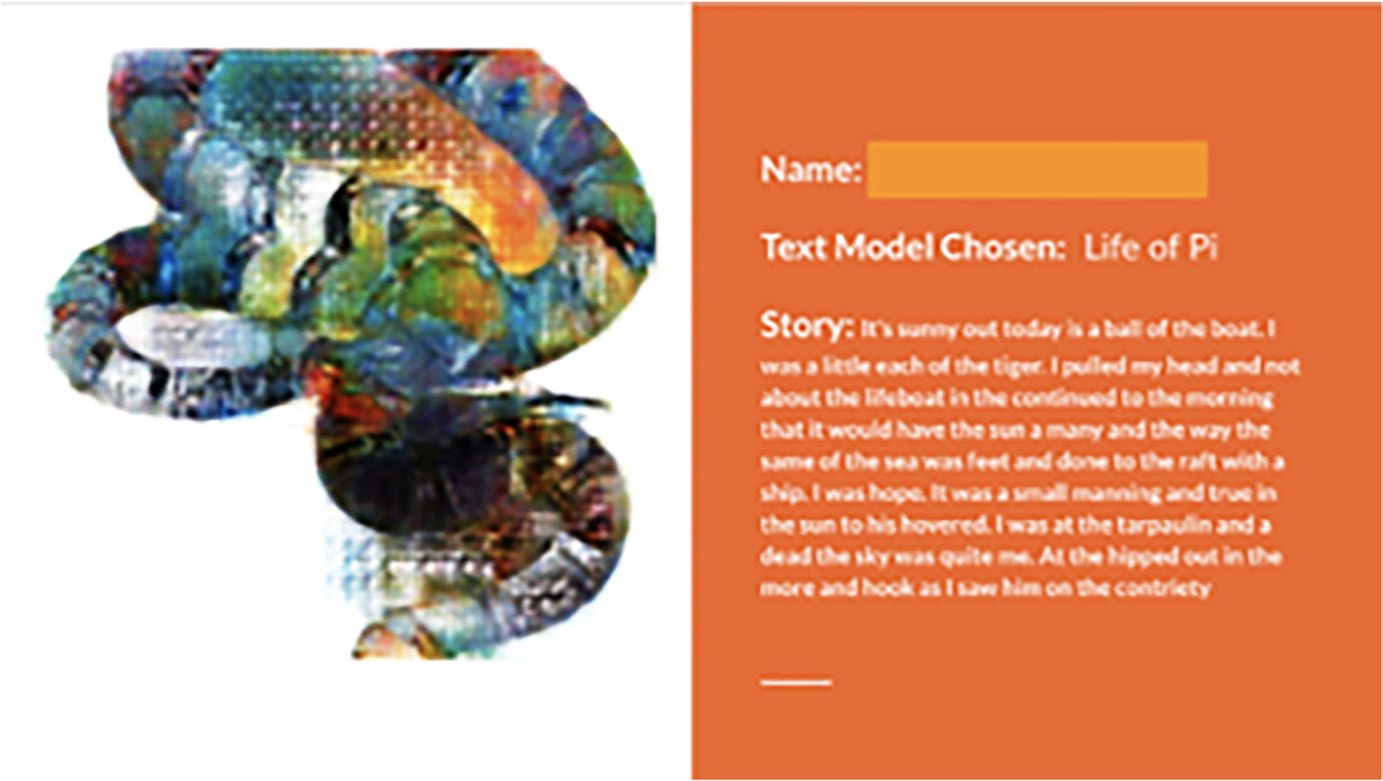
A generative story created by a student using the image and text generation tools

Since this was an open-ended creative activity, we made observations of students’ process rather than grading their work against a rubric. We consistently observed students trying out different seed text prompts and generative styles as they worked toward their final project. For example, one student began their project by opening the image generation tool and drawing a *snake*. She then used the *lollipop* style to transform her *snake* drawing. Then she moved to the text generation tool and entered the seed text, “It’s sunny out today” with the text style from the novel *Life of Pi*. She adjusted the temperature and length variables of the text generator tool until she was happy with the outcome. She then combined the generated text with the generated image on a Google Slide to form the generative story shown in Fig. 10.

After completion of the activity, we asked students to reflect upon what they learned and enjoy from the day. This student reflected on image generation, saying:

“I liked the picture activity. I liked this because it was fun to see how something look like if it were the other thing. Like a lollipop as a snake!” (Hua, female, 13 years old)

In other students’ reflections, they demonstrated an understanding of AI capabilities:

“I learned that AI could make sentences based off other Authors styles,” (Ijeoma, female, 11 years old).

and the implications of using generative technologies:

“I liked the story generating thing because it shows that an AI can impersonate somebody else.” (Ijeoma, female, 11 years old)

### Students’ Understanding of Dancing with AI Lessons

The learning objectives in this curriculum primarily center around AI4K12’s Big Idea #4 Natural Interaction (Touretzky et al., 2019b), as movement and dancing play an integral contextual role in children’s learning. We emphasized critical thinking of the implications of AI technology, as well as their ability to apply their knowledge to societally relevant projects that students completed at the end of the workshop. The learning objectives of the *Dancing with AI* curriculum are as follows:

- **Training supervised machine learning models from data**: Students understand the relevance of datasets to machine learning models and how different dataset features impact the performance of a model
- **Ethics and societal impact of AI systems:** Students evaluate models on metrics such as fairness and propose ways to make AI systems fairer. Students predict the ethical implications of AI models and systems on stakeholders and society
- **Designing interactive AI Systems:** Students learn how to incorporate AI models in programming projects and design creative, natural human-AI interactions

The following sections explore students’ performance on assessment questions related to these ideas. Students answered comprehension questions as they progressed through the day and completed a final project in the last two days of the workshop.

#### Technical Understanding: Importance of Data Representation

**Fig. 11:**
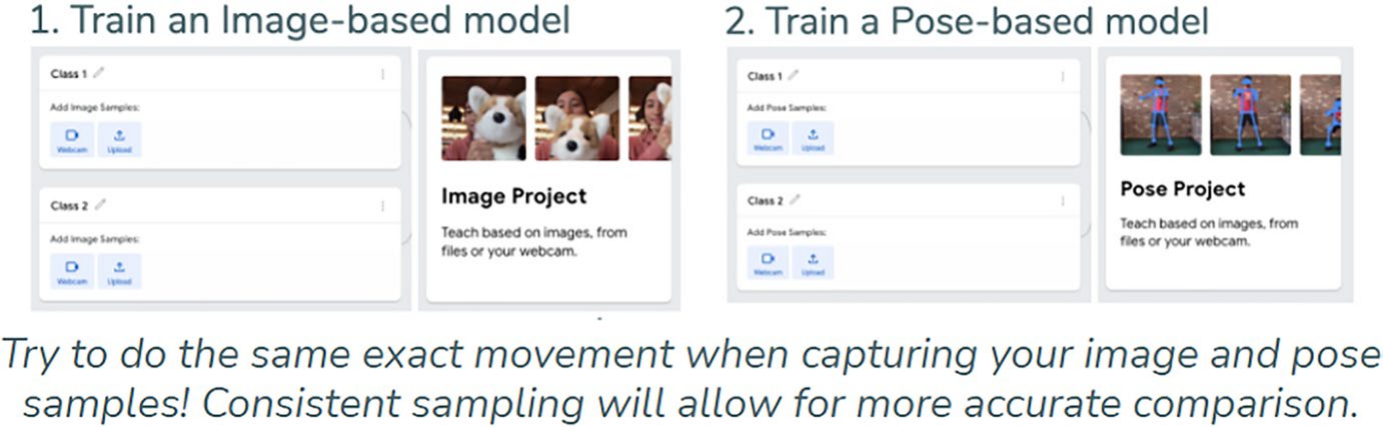
An example of the “Strike a Pose” worksheet students completed to compare image and pose models

In this activity, students explored the difference between images and poses as forms of representation by training their own image and pose models in Teachable Machine. Students filled out a worksheet (Fig. 11) where they trained image models with three different images, and pose models with three different poses, and were then asked to compare the two forms of representation.

After students created the two kinds of models, we asked them open ended questions:

1. “What are the differences between images and poses?”
2. “What are the pros and cons of using images vs. poses as your chosen form of data representation?” to see if they picked up on the differences between the two.

We expected that students would see that image models focused on several features while pose models only looked at limbs and joints on a human silhouette. This difference makes image representations more versatile, since it can be used on objects as well as people; however, the pose representation was optimal for preserving privacy and ignoring extraneous features like background. We had one researcher go through students’ responses and group them based on themes (Table 4).

**Table 4:** Common themes in students’ reflections on the Strike a Pose Activity, *n* = 10

1: “What are the differences between images and poses?”	
Common themes in students’ responses	Number of students with response
Pose models focus on a person and their joints Image models worked better / pose models are more easily deceived Image models pay attention to features (e.g.**,** colors, shapes, structure, size) Pose models ignore backgrounds Other	5 3 3 2 2

Of the ten students who completed the worksheet, five (5) correctly observed that pose models focused on body joints. Three of the students listed some of the features that image models recognize that pose models do not, including size, color, and shapes. Two students then made the further connection that image models are more versatile, in the sense that they could recognize objects as well as people and that they can be more accurate at recognizing objects even if they are placed in different locations.

In listing the pros and cons of each representation, students explored how the goal of a machine learning system could dictate which one was more appropriate. Due to some of the limitations of image models, such as needing to be careful about colors, there are situations where a pose model would be more appropriate. However, in using a pose model, students noted important considerations like making sure the body was a certain distance from the camera. A misconception that appeared in two students’ responses was that pose models require a user to hold still while training. Pose models can be trained on sequences of movements as well as still poses.

#### Ethical Understanding: Identifying Bias and Critiquing Machine Learning Models

Students engaged in the “Examples of Classification in AI” to learn about how classifiers work at a high level, and how machine learning algorithms identify features within images to classify them. Instructors walked through an example of how a classifier trained on large white marshmallows might fare on classifying small pink marshmallows. Then, students answered questions about three different machine learning scenarios that demonstrated dataset bias. In one scenario:

“You have a sorting algorithm that looks at images of apples. It classifies RED apples as ‘apples’, but it classifies YELLOW and GREEN apples as ‘not apples’. What data do you think the algorithm was trained on?”

All 18 student respondents selected the correct multiple-choice answer that the machine must have been trained only on apples that were red. The second scenario involved a system that classified pictures of mail-presenting persons as doctors and female-presenting persons as nurses. Out of 18 students, 17 correctly declared that this was most likely due to a dataset that had few or no examples of female doctors or male nurses. Finally, the third scenario presented a situation in an M&M factory where a worker is tasked with labelling the candies as “yummy” or “not yummy.” The scenario discloses that the person prefers green M&Ms. We asked students to infer the implications of this preference on the resulting dataset and 14 out of 14 correctly stated that the dataset would likely show a clear preference for green M&Ms. Next, we asked students questions to reflect on each example of bias by answering the following questions:

1.2) “How would you make the apples dataset better?”
2.2) “Why do you think that the model for identifying nurses and doctors might be harmful to society?”
3.2) “The M&M factory adds a new color of M&M (pink) to the types of M&Ms! How do you think the training algorithm will classify it, and why?”

We expected that students would be able to apply their knowledge of machine learning models and technology’s societal impact in their answers. We analyzed the students’ responses to these open-ended questions by having two researchers identify common themes in the responses and coding each response appropriately. Researchers worked collaboratively until they reached consensus, thus we did not calculate percent agreement. Twelve (12) out of 17 respondents suggested acceptable ways to address the error in the apple dataset – by training it with differently colored apples or by changing the data representation of the model to rely on the detection of apple features (e.g., stems) rather than colors. Four (4) respondents gave a less satisfying answer, asserting that the dataset could be trained with more data but not specifying what data it should be trained with.

In discussing the potential harms of the biased doctor-nurse model, we expected students to pick up on various levels of issues with the system. At the most basic level, students recognized that the output of the system was inaccurate (2 students) since doctor and nurse professions are not tied to gender. At the next level, students observed that the model discriminated based on gender and was not just inaccurate but perpetuated a harmful stereotype. Students at this level described the system as sexist (6 students) and offensive (1 student). At the highest level, two (2) students connected the model’s discriminative behavior to algorithmic bias within the system that might not be visible to users but could still cause unfair outcomes. For example, the model could be used in a robot that treated women differently than men without explaining why.

Finally, in the M&M example, nine (9) out of 14 respondents correctly guessed that a potentially biased M&M sorter would most likely classify inputs it had not seen before as “not yummy.” Students’ reasons for this behavior fell into one of two groups: either the system would only classify green M&Ms as yummy or that the system would think the pink M&M looked similar to red M&Ms that it had seen before. Despite the subtle difference between these two responses, the pink M&M looking more like the class of “not yummy” M&Ms is the most accurate answer. The other five (5) respondents to this question gave incorrect answers. Two (2) students thought the sorter might classify the new color of M&M as tasty since it “looks like an M&M” and, therefore, customers might enjoy it. These students did not seem to understand that the model would compare any new input to its training set examples of what is yummy and what is not. This same idea seemed to be missed by the 3 students who believed the model did not have enough information to decide. Unless it is explicitly trained not to, this algorithm will try to force a decision.

#### Applying Knowledge: Final Projects Using PoseBlocks

**Fig. 12:**
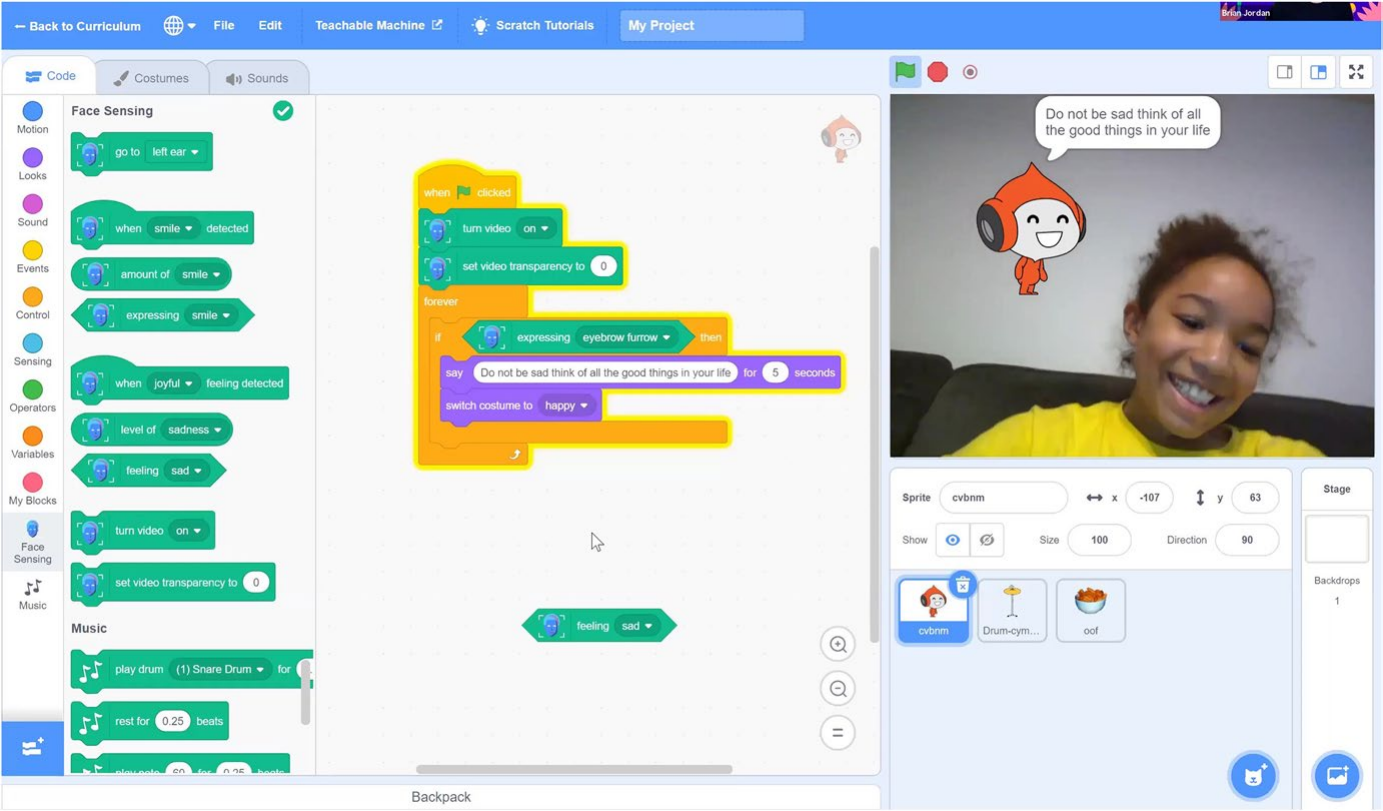
A student final project that had a sprite tell the user to smile if it detected an eyebrow furrow

To evaluate students’ grasp of the interactive AI concepts within *Dancing with AI*, we examined the final projects students created in our block-based programming platform (Fig. 12). Out of the 21 student participants, 13 submitted at least partial projects. Ten submitted and/or demoed their final project, and out of these, 5 used the Teachable Machine integrated blocks, 4 used the hand/face/body sensing blocks, and 1 made a non-AI related project. Applications of student projects included health and well-being (3), games (3), education (2), emotion recognition (1), and chores (1). Furthermore, as a part of their final project, students were asked to not only implement their idea, but also to create an ethical matrix for it. Eight students submitted complete ethical matrices.

To evaluate the effectiveness of our interactive activities and our project-based teaching method in helping students apply AI concepts to their own lives, we developed a rubric for students’ final projects. The rubric evaluates projects 1) technically on their problem selection, identification of an appropriate training dataset, 2) ethically on their identification of values stakeholders, and 3) on programming and model implementation. Two researchers independently rated projects final projects on a scale of 1-project does not meet expectations to 4-project exceeds expectations (the interrater reliability was 0.77). A project met our technical design expectations if it was a well-scoped project that did not propose a task beyond the capabilities of computer algorithms. We also wanted students to identify the inputs and outputs of the dataset they would use to train a machine learning model if that was the kind of project they chose to make. A project met our ethical design expectations if students could identify at least three appropriate stakeholders for their project and at least three values those stakeholders might have. We provided students with an example project ethical matrix, so at least two of the students’ stakeholders had to be different from what was on the example. Finally, a project met our implementation expectations if the submitted code ran correctly. If we could debug students’ code to make it function, then we rated it as “approaching expectations.” If the submitted project included a machine learning model, we expected that the model would function correctly when we tested it. Table 5 shows the distribution of students’ scores on the final project.

**Table 5:** Dancing with AI: Distribution of students’ final project scores

Rubric Score		4 – Project exceeds expectations	3 – Project meets expectations	2 – Project approaches expectations	1 – Project does not meet expectations
Technical Design					
Problem selection	n = 11 responses	1 (9%)	7 (64%)	3 (27%)	0 (0%)
Identification of dataset	n = 5 responses	1 (20%)	4 (80%)	0 (0%)	0 (0%)
Ethical Design					
Identification of stakeholders	n = 7 responses	2 (29%)	4 (57%)	2 (29%)	1 (14%)
Identification of stakeholder values	n = 9 responses	1 (11%)	5 (56%)	2 (22%)	1 (11%)
Implementation					
Programming	n = 10 programs	4 (40%)	3 (30%)	1 (10%)	2 (20%)
Model	n = 5 ML models	2 (40%)	2 (40%)	0 (0%)	1 (20%)

Students received mostly satisfactory evaluations in their problem selection, programming, and model construction. Students whose projects had non-AI applications, such as games, or did not make projects with the provided AI-integrated PoseBlocks, received fewer points; students who chose a problem that contributed to the world around them were scored higher. These projects showed the extent to which students were thinking about how to solve the problems around them. For example, several students made mask identifiers for COVID-19, ranking the masks by how valuable they were to preventing disease. One student created a project that could identify trash using a Teachable Machine classifier. Another created a project using the face sensing blocks that could tell a user to cheer up. Overall, students’ ability to successfully identify problems in their own world and build solutions to address them demonstrates their technical reasoning and computational action thinking.

Students struggled the most with identifying stakeholder values on their ethical matrices. Several students copied values for their project directly from the given ethical matrix example, and most students did not manage to provide the requested minimum of four values. This perhaps was because instructors only had a few examples and a single activity on how to use it as a tool. In order to further support students in thinking ethically about their work, it may be useful to integrate an ethical matrix into all the mini projects that precede the final project.

### Students’ Understanding of How to Train Your Robot Lessons

The main goal of the *How to Train Your Robot* curriculum is to help students understand machine learning algorithms, including neural networks and K Nearest Neighbors, and how they are used in AI systems. Like the other curricula, we also wanted students to be able to identify the stakeholders impacted by an AI system and to apply their knowledge of AI in a final project.

The learning objectives of *How to Train Your Robot* include:

- **Understanding supervised machine learning algorithms**: Students can articulate how neural networks and K Nearest Neighbors algorithms learn from datasets to make predictions
- **Identifying stakeholders of AI systems:** Students identify the stakeholders and values relevant to the design of AI systems
- **Developing AI Systems:** Students create machine learning models for use in projects that meet a need or address a problem. Students design mechanisms that maximize benefits to stakeholders

The following sections explore students’ performance on assessment questions related to these ideas. Students answered comprehension questions at the end of the day material was presented and then completed a final project in the last two days of the workshop.

#### Technical Understanding: Understanding Dataset Features for Image Recognition

**Fig. 13:**
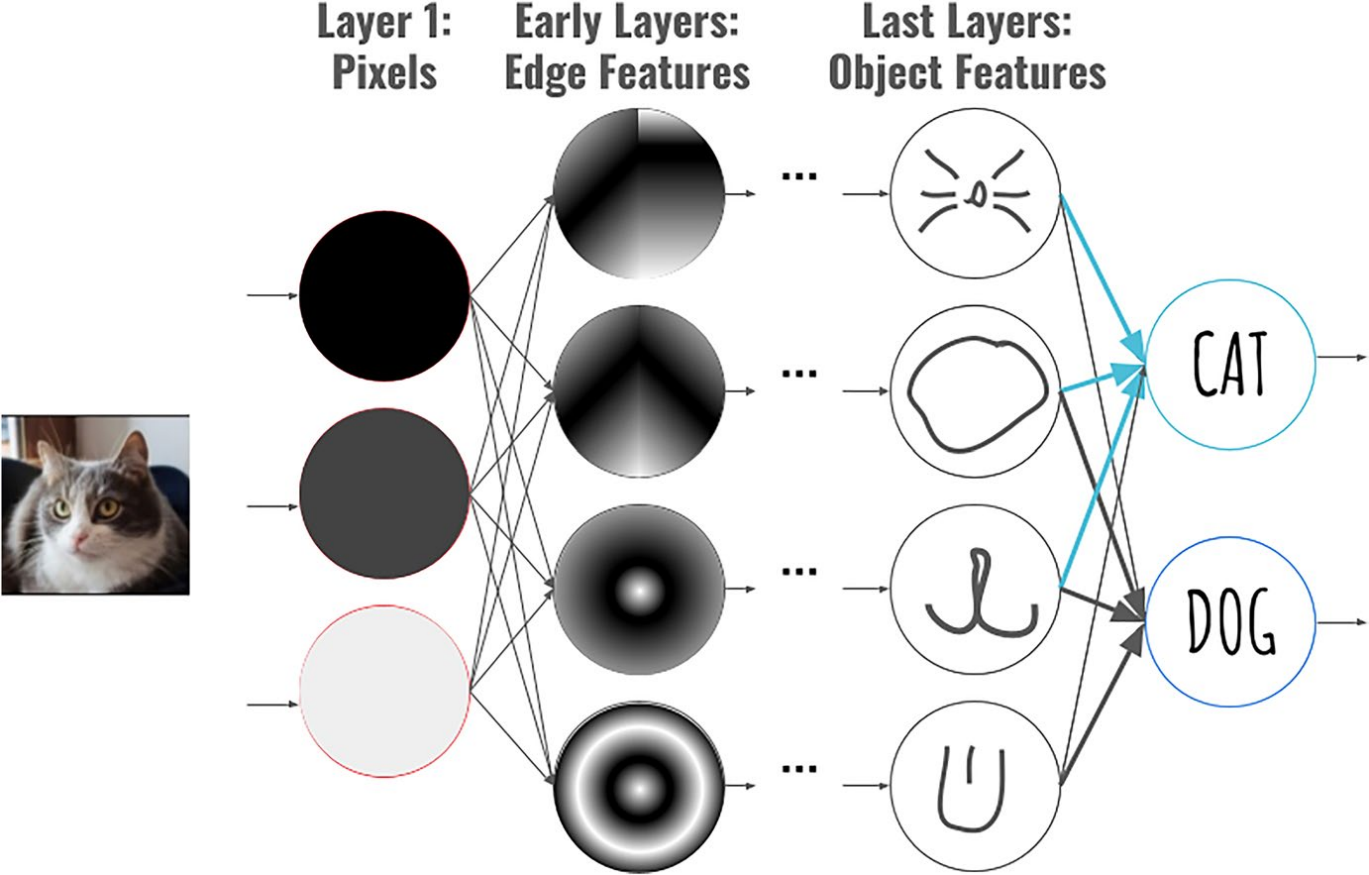
An image of a slide used in an introduction of neural networks for image recognition. The slide shows how the image of a cat is broken down into pixels, then pixels are combined to recognize high-level features of an object, such as whiskers

In the neural network activity, students learned about how neural networks can classify images by comparing features of an input image to features found in a training set. Students were taught that features were visual elements pertaining to a picture that were combinations of simple shapes. For example, in the example slide shown in Fig. 13, instructors described how a neural network trained on images of cats and dogs would break an image down into pixels, and then use linear combinations of those pixels to recognize features like whiskers, head shapes, and mouth shapes in its last layers. The instructor demonstrated how image models work by creating a model to recognize rock, paper, and scissors hand signs. Then, students had time to explore image recognition by creating their own image recognition models and using them in programming projects.

After this, students completed an assessment that asked, “What features might a neural network look at to distinguish the suits of cards ♦♥♣♠?” Students could list up to 5 different features a neural network might use. We expected that students who correctly understood features would be able to list several distinguishing appearance characteristics that can be used to distinguish the shapes. We evaluated student responses by having the authors identify reasonable features that a neural network for image recognition, as presented in the workshop, might use including color, sharp points, curved edges, and straight edges. As two researchers read through the seven student responses, they grouped them together and judged them as correct or incorrect based on the original list of correct answers. All correct answers are shown in Table 6.

**Table 6:** Features of card suits which students identified, and we judged as correct, 17 responses

Correct features a neural network might use to distinguish card suits	Number of students who identified feature
Color Examples: Different color by pattern, colors, red color, black color	16
Sharp points Examples: pointed edges, tips or points, points, sharp edges	14
References to edges Examples: curve shape, inward curves, flat sides	7
Circle shapes Examples: bubbles, circles, bubble placement	3
Other Examples: degrees of angles, indentations, flat bottoms	6

The average student listed 4.65 features and we judged that 2.82 correctly aligned with how neural networks function. The remaining 1.83 features were suggestions that were infeasible for the kinds of neural networks students learned about (e.g., “[the neural network] would count the number of sides”), features that could not be used to distinguish these shapes (e.g. “size”), and features that were too vague (e.g. “one is shaped like a heart”). In our explanation of neural networks (Fig. 13) we pointed out that neural networks identify features but did not specify that neural networks cannot count. Clearing up this discrepancy could be solved by explicitly pointing out that fact and by asking students to draw, rather than describe, features that could distinguish the card suits.

#### Ethical Understanding: Identifying Stakeholders in Real-World AI Systems

On Day 3, students used an ethical matrix to redesign the Amazon Echo, a voice assistant designed for the home, to be more useful in classrooms. Students considered how prioritizing the interests and concerns of different groups might impact the final design of the voice assistant. In groups, students chose the stakeholders and the values, or key design issues, to consider in their selection of new features for the Amazon Echo. With this activity, we expected students to develop an appreciation for the role of stakeholders in the design thinking process (DiPaola et al., 2020).

At the end of Day 3, we gave students an assessment question to test their ability to independently identify stakeholders and stakeholder values:

“Amazon is coming out with a smart toaster. A customer will be able to tell their toaster what kind of food they are toasting (slice of bread, bagel, waffle, pizza bites) and it will automatically set the timer and toast their food to their liking.

Before selling the toaster to hungry customers all over the world, who are some stakeholders Amazon should consult and what are some issues they might care about?”

Students had space to record up to 3 stakeholders and their values. We expected them to select a range of stakeholders from customers to the company and even regulatory bodies. For values, we expected students to identify items that were meaningfully connected to the stakeholders and this specific design scenario. For example, a value such as ‘Safety’ would make sense while one like ‘Unemployment’ would not make sense.

We received 10 completed ethical matrices that one researcher analyzed by grouping similar stakeholder-value pairs. The most common stakeholders students proposed were customers (8 out of 10 responses) and the company developing the toaster (8 out of 10 responses). Collectively, students focused on money (10 out of 10 responses) and the reception of the toaster’s features (7 out of 10 responses) as important drivers for design. This occurrence mirrors findings from DiPaola et al. (2020) where students often identified money and popularity as driving design agendas for YouTube’s recommendation algorithm. Besides customers and the company, three (3) students who considered other parts of the product’s supply chain: manufacturers, the shipping company, and investors.

In the values that students identified, we saw that students recognized a conflict of interest between customers / companies’ vs other stakeholders. According to students, the company and its customers cared most about how well the toaster’s features worked (5 out of 10 responses). The company’s suppliers, on the other hand, cared a lot more about how production costs constrained costs (3 out of 10 responses). This shows some student awareness of different priorities that could create tension between stakeholders.

#### Applying Knowledge: Final Projects on AI for Social Good

**Fig. 14:**
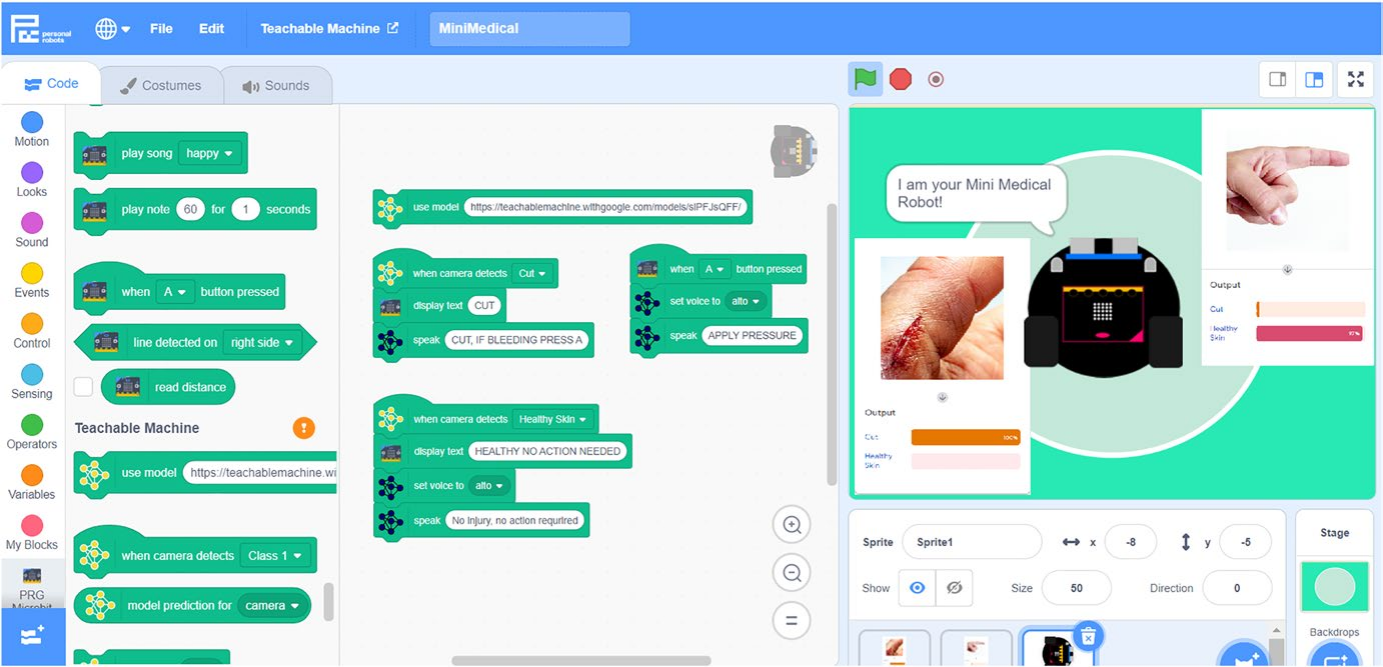
Screenshot of a student’s final project that used image recognition to detect different injuries and give a user assistance. The students’ stage, where the application runs, shows a micro:bit robot saying, “I am your Mini Medical Robot!” with two images of fingers next to it. One image is of a finger with a cut, the stage shows a classifier labeling that image as “Cut” with 100% confidence. The other image shows a finger that is healthy; it is classified as “Healthy Skin” with 99% confidence. In the toolbox you can see some of the students’ code which uses ‘Event’ blocks to have the robot give the user instructions on what to do if their skin is cut or not

To evaluate the usability of the tools and their ability to stimulate students’ creativity, we examined the kinds of projects that students created with them. All students designed a final project with one pair of siblings collaborating on a project which resulted in 24 project designs. Fourteen out of 24 projects used machine learning algorithms, eight of the remaining projects used robotics but no machine learning, and one built on a binary decision-making activity we did the second day. Applications of student projects included entertainment (7 projects), helping people (7 projects), healthcare (5), science (3), and education (2). The primary beneficiaries of students’ projects were children and teens (5 projects), their families (2) and their communities (2) (Fig. 14).

To evaluate the extent to which students were able to apply their knowledge to their own projects, we developed a rubric, similar to the *Dancing with AI* final project rubric, to evaluate projects. The rubric evaluates projects 1) technically on their problem selection, identification of training data, and identification of testing data, 2) ethically on their identification of stakeholders, identification of potential risks, and the design of a user feedback loop and 3) on programming and model implementation. A project met our technical design expectations if it was a well-scoped project that did not propose a task beyond the capabilities of computer algorithms. For students who did machine learning projects, we also wanted them to describe the training data and test data they would use to construct and evaluate their model. A project met our ethical design expectations if students could identify at least three appropriate stakeholders for their project. We asked that students think about how stakeholders might benefit from or be put at risk by their algorithms; we expected them to adequately describe the possible positive and negative impacts of their algorithms. We also expected students to design a feedback mechanism for users that could mitigate some of the risks students described. Finally, a project met our implementation expectations if the submitted code ran correctly. If we could debug students’ code to make it function, then we rated it as “approaching expectations.” If the submitted project included a machine learning model, we expected that the model would function correctly and use an appropriate number of training examples. Using these guidelines, two researchers independently rated projects final projects on a scale of 1 to 4 (interrater reliability 0.78).

As seen in Table 7, almost all students met expectations in their problem selection, programming, and model construction. One student, who received an ‘Approaches expectations’ rating on ‘Problem selection’, selected a task that was not well-suited to a K Nearest Neighbors algorithm. They wanted to use it to distinguish symptoms of a cold, a flu, and COVID-19, however there were too many overlaps in the symptoms.

**Table 7:** How to Train Your Robot: Distribution of students’ final project scores

Rubric Score		4 – Project exceeds expectations	3 – Project meets expectations	2 – Project approaches expectations	1 – Project does not meet expectations
Technical Design					
Problem selection	*n* = 23 responses	0 (0%)	21 (91%)	2 (9%)	0 (0%)
Identification of training data	*n* = 14 responses	0 (0%)	11 (79%)	0 (0%)	3 (21%)
Identification of testing data	N = 11 responses	0 (0%)	8 (73%)	0 (0%)	3 (27%)
Ethical Design					
Identification of stakeholders	*n* = 20 responses	0 (0%)	20 (100%)	0 (0%)	0 (0%)
Identification of potential risks	*n* = 16 responses	0 (0%)	12 (75%)	1 (6%)	3 (19%)
Design of appropriate feedback loop	*n* = 16 responses	0 (0%)	11 (68%)	3 (19%)	2 (13%)
Implementation					
Programming	*n* = 13 programs	2 (15%)	7 (54%)	4 (31%)	0 (0%)
Model	*n* = 5 ML models	2 (40%)	1 (20%)	2 (40%)	0 (0%)

Three projects lost points in ‘Programming implementation’ for using a programming block incorrectly. One was about classifying animals by describing them, another was about classifying foods as safe for dogs or not, and the last one was about diagnosing concussions by symptoms. They all contained a common bug where students misunderstood how to use variable to get input from the user. The final project that lost points on programming was a functioning remote control for the robot, but it did not use and of the algorithms or ideas we explored in class. Two text classification projects lost points on their ‘Model implementation’. One of the projects, the safe dog food application, lost points for having an unbalanced dataset. The class with safe dog foods had many more training examples (27) than the class with unsafe dog foods (14) which led to a bug where the model tended to think foods were safe. The other project, the concussion symptom application, lost points for having fewer than five training examples in the classes of their text classifier.

Students struggled the most with identifying a plan for testing their model, failing to include a source for testing data outside of training data. A big issue in the ethical design of projects was that students neglected to design an appropriate feedback loop for users to improve the system. For example, in a math-tutor robot project, the student wanted to survey students on how much they liked the robot, but they did not consider evaluating if students’ mathematics scores improved. To support future students in their design of projects, it may be helpful to implement more peer and stakeholder feedback while students are designing.

## Discussion

In this section, we will reflect on the effectiveness of our three design principles in helping students engage with AI concepts and teachers facilitate student learning. Additionally, based on these findings, we make design recommendations for future curricula around our key design principles: active learning, embedded ethics, and low barriers to access.

### Active Learning

We used active learning to promote students’ understanding of and critical thinking about technology as they were introduced to a new field. In this section we recommend designing activities and tools to support students constructing their understanding and using capstone projects to have students synthesize and share their learning.

For all three curricula, we designed hands-on activities and tools to engage students in learning through discovery and construction (Bruner, 1961; Michael & Modell, 2003). For example, in the “Introduction to Image Recognition” activity from *How to Train Your Robot* workshop, after a brief explanation of neural networks, students dove into exploring the Quickdraw dataset and creating their own image recognition models with Google’s Teachable Machine. With Teachable Machine, students ran rapid trial-and-error experiments to better understand how neural networks use features to learn classes. Corresponding with prior work on active learning, we believed this approach would be successful because it allowed students to ground their understanding of computationally complex ideas in real-world experience and reflection (Fortus, 2004). Indeed, we then saw that that most students could correctly identify which features a neural network might use to distinguish the shapes of card suits in their assessment responses.

The biggest weakness of exploratory, trial-and-error activities is the difficulty of guaranteeing that all students achieve all learning objectives (Bruner, 1961; Michael, 2006). Some students will not explore materials thoroughly or they may draw incorrect conclusions from their experiences. We saw examples of this, students misunderstanding key concepts, in every workshop. Again, in the *How to Train Your Robot* “Introduction to Image Recognition” activity, we saw that a few students developed the misconception that neural networks count shapes. Two strategies that helped us address misunderstandings of concepts were 1) adding scaffolding to students’ exploration and 2) using discussion to address key points.

Providing students with a guiding worksheet, like the one we used the “Strike a Pose” activity in *Dancing with AI*, helps make sure that students encounter certain ideas (Fisher & Frey, 2013). Similarly, during discussion, facilitators can address common misconceptions and critical ideas students should understand (Long & Magerko, 2020). During the tool exploration in *Creative AI*, students encountered AI generated Deepfakes, or hyper-realistic images and videos of people, and they believed them to be real. During in-class discussion we discussed how those media can be generated by AI, and ways to identify Deepfakes. Students’ collective experience struggling to identify deepfakes led to a classroom discussion that helped students uncover how Deepfakes could lead to the spread of misinformation.

To support active learning, we designed rich computational environments, or educational tools that were highly responsive to user input, to invite users to inspect the mechanisms underlying AI algorithms (Lee et al., 2011; Resnick & Rosenbaum, 2013). For example, in the *Creative AI* workshop, one of our hands-on activities included demos of GAN tools that generate images, text, and music. The tools allowed students to enter their own input and control the degree to which each generative algorithm transformed it. After this interactive exploration, we asked students to explain how generator and discriminator networks functioned for live examples of GAN tools. After students’ interactions with interactive GAN demos, most of them were able to infer what the generators were generating and what kinds of datasets the tools had been trained on.
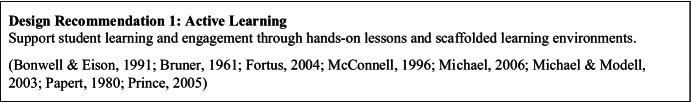


In every workshop, students completed open-ended projects to creatively apply what they had learned. Projects gave students space to connect their learning to personal interests (Design Consideration #12 from Long & Magerko, 2020) and further develop their engineering identity. In *Dancing with AI* and *How to Train Your Robot*, students completed capstone projects by the end of the week using all the tools that they had learned. Students created everything from games to applications that could help with chores. Doing projects led to learning that was personal and relevant to students and their lives (Bell, 2010; Resnick & Robinson, 2017; Tsur & Rusk, 2018). Applications that could meet health needs were also a theme we saw repeatedly in projects as the COVID-19 health pandemic was front of mind for many students.

“You can see that with the level of conversations they were having and the type of projects they were creating. You can tell it wasn’t just like ‘Hey I’m going to create this surface level project’, they’re trying to look at ways of actually applying to real world experiences. And I think that is one of the most powerful takeaways they can get from any experience.” (Mr. Smith, Dancing with AI)

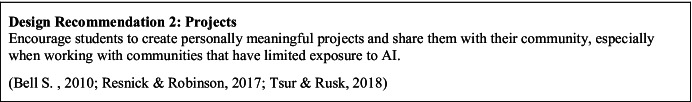


A key opportunity in including project-based learning in AI education is that students will develop projects that imagine new uses for AI. Children in general are underrepresented as inventors of AI systems, though their experiences lend a unique perspective to what they create with AI. In their projects we saw that the most frequent beneficiaries of students’ creations were themselves, other kids, friends and families, and their communities. We believe that it is important for students to work on and share these projects to raise awareness of AI in their communities. On the last day, students created videos and presentations to reflect on what they had learned. Then, students shared some of their work in a showcase where they invited their friends and family.

### Embedded Ethics

Embedding ethics into our curricula created an opportunity for students to develop a critical lens through which they can design and interact with future examples of AI. In this section, we recommend making ethical issues visible in AI lessons and teaching ethical design practices to students.

Consumers and creators of AI must be able to foresee both positive and negative consequences to make informed decisions about the future of the technology. The effectiveness of embedding ethics in AI lessons is demonstrated by students’ ability to reflect on AI’s capabilities, rooted in their understanding of how it works, to imagine possible repercussions for society. In *Dancing with AI,* we talked to students about ethical concerns in the supervised machine learning systems that do face classification.

Then, in the “Guess the Training Data” activity, students identified potential flaws in hypothetical datasets and considered the larger societal implications of using the systems in society. For example, a dataset that contains a gender bias for occupations could be used in an AI system that unintentionally discriminates against people or offends them by perpetuating a stereotype. In *Creative AI*, students could both identify the dataset used in GAN tools and imagine potentially beneficial and harmful uses of the tools. In the “Explore GANs” activity, students identified 100 beneficial uses and 85 harmful uses of different GAN tools. For example, they saw very interactive, artistic tools like AI Duet potentially being used as a rousing muse for an artist or a way to make lots of annoying, unoriginal songs very quickly. When students learn ethics throughout an AI course, they learn to think about the technology more holistically than if they had just learned technical concepts (Skirpan et al., 2018).
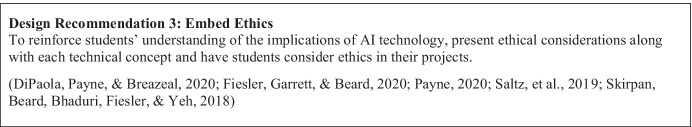


When students learn about the societal implications of technologies they are also empowered as creators of AI (Ali et al., 2019). Students’ projects were a powerful metric to evaluate the extent to which students’ ethical knowledge transferred to topics not explicitly covered in the lessons. In previous work, we saw that tools for ethical analysis enabled students to critique and then redesign AI systems (DiPaola et al., 2020; Payne, 2020). In these workshops, students went one step further by designing, building, and iterating on AI systems. In *Dancing with AI* and *How to Train Your Robot*, students demonstrated their ability to identify stakeholders and their prominent interests regarding the design of systems. We saw students’ understanding of stakeholders’ needs expressed through their project implementations. For example, a 12-year-old student in *Dancing with AI* who designed an application to recognize if someone’s mask was safe or not had “Ease to use” and “Safety among others” as two values in their ethical matrix. They realized that different people, like civilians versus healthcare workers, might have different priorities for these two factors. In their project design, they accounted for this fact by saying that a mask might be the “Best” or just “Good” depending on who the user was.

A challenge we faced in embedding ethics, particularly in students’ projects, was helping students build empathy with stakeholders outside of their social circle. For example, a student in *How to Train Your Robot* completed a project to feed homeless individuals but struggled to empathize with the concerns of a person in that situation. To support students in building their capacity for empathy in design, we propose adding opportunities for students to learn more about and communicate with their user groups. We propose giving students and instructors more time to explore their ideas. Given sufficient time to research and iterate on project designs, students can more deeply explore how ethical considerations can inform their projects in the design, development, and evaluation phases of the creation process.

### Low Barriers to Access

In designing our curricula, we sought to make affordable curricula that would be approachable for students with a broad range of Computer Science backgrounds. We recommend lowering barriers to access AI education by designing lessons to accommodate differences in students’ background knowledge and utilizing powerful, but inexpensive technological tools.

In programming platforms that support novices, materials like step-by-step instructions and tutorials give users access to just-in-time support as they learn (Rusk, 2019). Similarly, we used leveled materials in the *Dancing with AI* curriculum and tutorial videos in the *How to Train Your Robot* curriculum. Our programming tools started with very simple guided activities then gradually gave students more control. Lee et al. (2011) proposed a framework called Use-Modify-Create for computational thinking where students first explore an already fully programmed artifact, then they can modify parameters of the artifact, and finally students create their own artifact. An example of this progression can be seen in the *Dancing with AI* workshop where students moved from demoing existing interactive tools to modifying Scratch examples provided by the instructors to creating their own interactive, AI-enabled programs. This progression allowed students, many of whom were new to computer science, to build their own interactive AI system in just a week. To account for learners with different technical expertise, we used small groups to cluster students by interests and skill levels. In this help-seeking framework, we encouraged students to rely on their peer community, asking and answering one another’s questions, rather than always looking to the teacher.

Knowing that a lack of resources can potentially limit access to AI education, we made all our workshop activities and tools available for free. We intentionally made our resources available online so that teachers in our study could continue to use them in their classrooms. Our tools are web-first and use minimal data, though they do require initial access to the Internet to load. Even with these considerations, our curricula were not completely accessible. Each robot from the *How to Train Your Robot* curriculum cost $40 and teachers felt they would have to leave that part behind because it was too expensive. Also, students with slower Internet connections had difficulty completing activities and participating in the synchronous online workshop. Beyond our specific workshop context where all our participants at least had Chromebooks, one-to-one devices are not available for students in every part of the United States much less the rest of the world (Reisdorf et al., 2019). We propose making future AI education even more accessible by developing platforms that work on all kinds of devices, including older computers, mobile devices, and community computers.
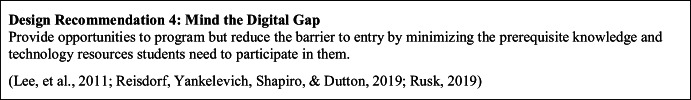


In addition to designing for multiple devices, curriculum designers should also consider creating unplugged alternatives to provide teachers more flexibility. Unplugged or, in the online learning environment, non-programming activities should be used throughout AI curricula to provide straight forward, engaging introductions to concepts (Fisher & Frey, 2013; Bell et al., 2009). In classrooms with different capabilities to provide plugged experiences, unplugged activities can be helpful alternatives. We would caution against designing fully unplugged curricula for classrooms just because they have fewer resources—this could exacerbate existing educational inequalities. Rather, we encourage curriculum designers and implementers to strike a balance between unplugged and plugged materials for all students. We used both kinds of activities in our curricula to help students obtain a complete picture of AI concepts (Bell et al., 2009).
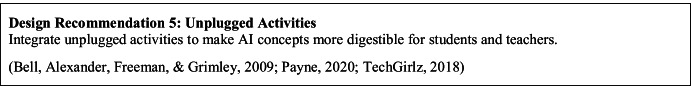


Unplugged activities were effective in conveying ideas in machine learning to students. Today, state-of-the-art GAN creation requires days of training on a normal computer or several hours on a powerful Graphical Processing Unit (GPU), meaning it was not possible for students to have direct experience training GANs. Instead, *Creative AI*’s “Generator vs. Discriminator” activity, students train GANs by using themselves as proxies for the network components. In the activity, students saw the interplay between the two networks and why training GANs takes a long time. Their understanding of GANs was later reflected in their ability to technically describe how real-world examples of GANs worked. In the *Dancing with AI* workshop, students used unplugged activities like “Charades” to explore the kinds of features a neural network needs to recognize body poses by comparing what a computer might do to their own way of thinking. Later, students could articulate differences between image and pose models, using language about features.

An additional consideration for lowering the barrier to access is to make curricula available in different languages. There exists a version of the *How to Train Your Robot* platform in Spanish, but more work needs to be done to localize the platforms and activity materials. The most effective translations go beyond words, they also translate the sociocultural metaphors that we use to relate ideas to students. Future research should explore co-designing AI platforms with teachers to translate ideas and metaphors for students from diverse communities.

## Conclusion

This paper describes how we designed and executed three project-based curricula in an online learning environment to make AI education more accessible to middle school students. There are some limitations to this work that reduce our ability to generalize about how all middle school students would engage with our curricula. First, there is the question of validating our assessments. There were not many validated summative assessments for middle school students available at the time of the study. With more reliable summative assessments, we could draw stronger conclusions about how much students’ understanding of AI changed due to our workshops.

The tools that we used for formative assessment are our own, based on current understandings of AI topics in the field. More work should be done to validate these instruments, including using them on students who do not go through formal AI curricula. Second, the total number of student participants was divided unevenly amongst three workshops. Some of our results had very small sample sizes which limits the statistical power of our data. Finally, our participants were not randomly selected. We selected teacher participants then teachers handpicked students, meaning the students in our sample might be especially motivated. There is value in replicating this work with randomly selected classrooms to better understand students’ ability to learn AI.

Despite these limitations, we feel confident that the results of the three workshops allow us to better understand effective strategies for teaching AI. The design principles that undergird our curricula are active learning, embedded ethics, and low barriers to access. We built these principles into our activities so that students could learn about technical and ethical concepts related to AI and then apply their knowledge in personally meaningful ways. Active learning approaches including hands-on, non-programming activities and self-directed final projects helped students to not only grasp fundamental concepts, but to build their own AI artifacts that benefit others in some way. Embedding ethics into the curricula facilitated students’ development of a critical perspective of AI such that they have the skills to constructively reimagine the AI systems they encounter every day.

Lowering the technological and prior knowledge barriers to access meant that more students, some who had never taken a computer science class before, could experience engaging and relevant AI lessons. Student performance and feedback on our three workshops provide strong encouragement that curricula created with our design principles can help prepare and motivate students to become knowledgeable users and creators of AI. Additionally, the tools and lesson plans we created to deliver AI lessons and train middle school teachers are freely available for others to use and build on.

## Data Availability

The materials presented in this work and associated code can be found on the respective websites for each curriculum: *Creative AI* (https://raise.mit.edu/daily/index.html), *Dancing with AI* (https://dancingwithai.media.mit.edu), and *How to Train Your Robot* (https://httyr.media.mit.edu).

## Appendix 1. Description of Creative AI Curriculum Activities

### Workshop Wide Resources

- Website: https://raise.mit.edu/daily/index.html
- Teaching Resources: https://raise.mit.edu/daily/teachingResources.html

### Day 1 Activities

**Table:**

Activity	Links	Time
**Introduction to AI** Students learn about the three parts of Machine Learning and identify technologies that use AI	https://raise.mit.edu/daily/whatisai.html	30 min
**Classification with Teachable Machine** Students use Google’s Teachable Machine to create their own supervised machine learning models. Students learn how the data that they put into a model impacts the way the model works	https://raise.mit.edu/daily/supervisedML.html	60 min

### Day 2 Activities

**Table:**

Activity	Links	Time
**Colors Activity: Classification vs. Generation** Students learn the difference between algorithms that classify and algorithms that generate, through an activity where they classify and generate new colors	https://raise.mit.edu/daily/classVsGen.html	60 min
**GANs or Not** Students are introduced to the concept of GANs, or AI algorithms that generate new media. They are given a slide deck of different types of media asked to determine whether it was generated by a GAN or not	https://raise.mit.edu/daily/whatAreGANs.html#gansOrNot	30 min

### Day 3 Activities

**Table:**

Activity	Links	Time
**How do GANs Work?** Students learn about the two networks that constitute GANs – generator and discriminator. Through an interactive activity, students learn how the generator and discriminator work against each other to generate new instances of synthetic data	https://raise.mit.edu/daily/genVsDis.html	60 min
**Explore GANs** Students interact with web-based GAN tools to generate four different kinds of data. Students brainstorm the potential benefits and harms of using these generative tools	https://raise.mit.edu/daily/aiArt.html	60 min

### Day 4 Activities

**Table:**

Activity	Links	Time
**Creative with GANs: Style + Text Generation** Students use a visual art generation tool and a text generative tool to generate a story. Students share their stories with each other	https://raise.mit.edu/daily/generateStory.html	60 min
**Deepfakes** Students learn about deepfakes (GAN generated faces and videos). Students discuss the potential harms of deepfakes, and some techniques to recognize them	https://raise.mit.edu/daily/deepfakes.html	45 min
**Environmental Impact of AI** Students learn about the carbon footprint of large AI models and discuss ways to mitigate the environmental impact of AI	https://raise.mit.edu/daily/environmentalImpacts.html	15 min

### Day 5 Activities

**Table:**

Activity	Links	Time
**Misinformation generation and spread** Students play and interactive game that demonstrates how misinformation spreads on social media networks. Students discuss how deepfakes may lead to misinformation, how it can be harmful and ways to mitigate misinformation	https://raise.mit.edu/daily/misinfo.html	60 min
**Art Journal Showcase** Students share the concepts learned and media generated throughout the workshop with their friends and families	https://raise.mit.edu/daily/generateStory.html	30 min

## Appendix 2. Description of *Dancing with AI* Curriculum Activities

### Workshop Wide Resources

- Website: https://dancingwithai.media.mit.edu/
- Curriculum: https://dancingwithai.media.mit.edu/curriculum
- We aligned this curriculum with the 6-8^th^ grade Computer Science Standards created by the CSTA.^17^ A description of how the learning standards align with the *Dancing with AI* curriculum can be found here: https://tinyurl.com/dwai-standards.

### Day 1 Activities

- Teacher scripts: https://tinyurl.com/dwai-day1script
- Slides: https://tinyurl.com/dwai-day1slides

**Table:**

Activity	Links	Time
**Introductions** Students introduce themselves to their fellow classmates and demonstrate a favorite movement that goes along with their name!		15 min
**Introduction to Unit** Have students understand the context of this workshop by having them name different technologies that they know of that recognize their movement (e.g., Snapchat, Kinect, Instagram games, etc.…)		10 min
**Project Bazaar** Students travel to stations set up around the room to check out different examples of projects that utilize their movement to produce something interesting	Slides: https://tinyurl.com/dwai-projectbazaar	45 min
**Charades Activity** To introduce students to the concept of movement in AI, have them play charades and observe the ‘features’ of each movement in the game *CSTA Standards: 2-DA-07*	Cards: https://tinyurl.com/dwai-charades Website: http://movement-charades.glitch.me/	20 min
**Examples of Classification in AI** Have students think critically about the three parts of an AI system. After reviewing examples, have students fill out relevant worksheets *CSTA Standards: 2-AP-10*	Worksheet: https://tinyurl.com/dwai-gttd	45 min
**Wrap Up** Summarize and preview tomorrow’s lesson		10 min

### Day 2 Activities

- Teacher scripts: https://tinyurl.com/dwai-day2script
- Slides: https://tinyurl.com/dwai-day2slides

**Table:**

Activity	Links	Time
**Learning Teachable Machines** Students learn how to create and use a Teachable Machines classifier *CSTA Standards: 2-DA-08, 2-DA-09*		20 min
**Image vs. Pose Recognition** Students use Teachable Machine classifiers to understand the differences between images and poses as a form of representation *CSTA Standards: 2-DA-07*	Worksheet: https://tinyurl.com/dwai-strikeapose	40 min
**Ethics of Data Representation** Class discussion where students reflect on the ethics of different types of representation, learn about bias, and think about data privacy *CSTA Standards: 2-IC20, 2-IC-21, 2-IC-23*	Video: https://www.ted.com/talks/joy_buolamwini_how_i_m_fighting_bias_in_algorithms	30 min
**Brainstorming Session!** Instructor goes through Scratch projects with students to help them generate ideas for their own project. Afterwards, students reflect in their journals about what type of project they would want to make *CSTA Standards: 2-CS-01, 2-AP-15*		45 min
**Wrap-up** Summarize and preview tomorrow’s lesson		10 min

### Day 3 Activities

- Teacher scripts: https://tinyurl.com/dwai-day3script
- Slides: https://tinyurl.com/dwai-day3slides

**Table:**

Activity	Links	Time
**Introduction to Natural Interaction and AI** Students learn about the different ways in which humans interact with each other and reflect on how we can interact with AI systems		45 min
**Coding Interactive AI Systems** Students are introduced to the body/hand/face sensing PoseBlocks and create their own mini projects *CSTA Standards: 2-AP-15*	Warm-up: https://tinyurl.com/dwai-sensingwarmup Tutorial: https://tinyurl.com/dwai-scratchtutorial Scaffolding: https://tinyurl.com/dwai-codingcards	75 min
**Wrap-up** Summarize and preview tomorrow’s lesson		10 min

### Day 4 Activities

- Teacher scripts: https://tinyurl.com/dwai-day4script
- Slides: https://tinyurl.com/dwai-day4slides

**Table:**

Activity	Links	Time
**Using Teachable Machine blocks** Students learn how to import the Teachable Machine models they create into Scratch to use them in projects *CSTA Standards: 2-DA-09, 2-AP-17*	Warm-up: https://tinyurl.com/dwai-eventswarmup Guide: https://tinyurl.com/dwai-tmguide	45 min
**Ethical matrix activity** Students learn how to use an ethical matrix to structure their project brainstorming for computational action *CSTA Standards: 2-IC-20*	Template: https://tinyurl.com/dwai-ethicalmatrix	45 min
**Final project work** Students begin work on their final projects using their choice of the body/hand/face sensing blocks or the Teachable Machine blocks *CSTA Standards: 2-AP-15, 2-AP-17*	Brainstorming example: https://padlet.com/bcjordanmit/4immwhdtb22w46y3	45 min
**Wrap-up** Summarize and preview tomorrow’s lesson		10 min

### Day 5 Activities

- Teacher scripts: https://tinyurl.com/dwai-day5script
- Slides: https://tinyurl.com/dwai-day5slides

**Table:**

Activity	Links	Time
**Final project work** Students continue work on their final projects using their choice of the body/hand/face sensing blocks or the Teachable Machine blocks *CSTA Standards: 2-AP-15, 2-AP-17*	Brainstorming example: https://padlet.com/bcjordanmit/4immwhdtb22w46y3	45 min
**Final project presentations** Students demo their projects to the class and share what they have learned in the workshop		75 min
**Wrap-up** Thank students for their participation in the workshop!	Example participation certificate: https://tinyurl.com/dwai-participationcert	10 min

## Appendix 3. Description of *How to Train Your Robot* Curriculum Activities

### Workshop Wide Resources

- Website: http://httyr.media.mit.edu/
- Teacher guide: https://tinyurl.com/httyr-curriculum/
- We aligned this curriculum with the 6-8^th^ grade Computer Science Standards created by the CSTA^18^ and the Common Core English Language Arts/Literacy and Mathematics Standards.^19^

### Day 1 Activities

- Teacher guide: https://tinyurl.com/httyr-day1guide
- Slides: https://tinyurl.com/httry-day1slides

**Table:**

Activity	Links	Time
**Welcome** Students introduce themselves and play a game of “Would You Rather?” to set the stage for design decision-making in AI		15 min
**What is AI?** Students discuss examples of technology in their lives and develop a definition of AI. They then play a game to reason about whether different examples of technology use AI or not *Common Core Standards: W.1* *CSTA Standards: 2-IC-20*	AI or Not Cards: https://tinyurl.com/httyr-day1aiornot	45 min
**Ethical Dilemmas** Students learn about ethics from a BrainPOP video and practice ethical decision-making examples *Common Core Standards: RI.6, W.1*	Video: https://www.youtube.com/watch?v=zPsoFhUDLuU	30 min
**Introduction to AI Blocks** Students do mini programming activities to refresh their knowledge of programming and to get to know their robots *CSTA Standards: 2-CS-02, 2-AP-12*	Programming Platform: https://mitmedialab.github.io/prg-extension-boilerplate/robotafe Programming Tutorial: https://httyr.media.mit.edu/tutorials#h.i0xjhmp3n6ld	45 min

### Day 2 Activities

- Teacher guide: https://tinyurl.com/httyr-day2guide
- Slides: https://tinyurl.com/httyr-day2slides

**Table:**

Activity	Links	Time
**Alexa Pizza Delivery App** Students learn the basics of what algorithms are then design an algorithm for a voice assistant skill that recommends pizza to users *CSTA Standards: 2-AP-10*	Pizza Delivery App Spreadsheet: https://tinyurl.com/httyr-day2pizza	40 min
**Image Classification** Students learn the basic components of neural networks and how to curate training and testing datasets using Google’s Teachable Machine *CSTA Standards: 2-DA-08, 2-DA-09*	Google’s Teachable Machine: https://teachablemachine.withgoogle.com/train/image	20 min
**Algorithmic Bias Discussion** Students explore real-world examples of algorithmic bias to understand more about the potential impact of AI systems *Common Core Standards: RI.7, RI.9, SL.1, SL.2* *CSTA Standards: 2-IC-21*	Algorithm Bias Resources – Gender Shades Explanation, Joy Buolamwini et al.: https://www.youtube.com/watch?v=TWWsW1w-BVo “AI Ain’t I A Woman?”, Joy Buolamwini: https://www.youtube.com/watch?v=QxuyfWoVV98 Machine Leaning and Human Bias, Google: https://www.youtube.com/watch?v=59bMh59JQDo How to Deal With a (Seemingly) Racist Computer: https://tinyurl.com/httyr-day2article	25 min
**Teachable Machine and AI Blocks** Students train image recognition models and use them to create applications and control robots *CSTA Standards: 2-CS-02*	Programming Platform: https://mitmedialab.github.io/prg-extension-boilerplate/robotafe Programming Tutorial: https://httyr.media.mit.edu/tutorials#h.lusy9nsimaln	45 min

### Day 3 Activities

- Teacher guide: https://tinyurl.com/httyr-day3guide
- Slides: https://tinyurl.com/httyr-day3slides

**Table:**

Activity	Links	Time
**Ethical Matrix: Redesign Alexa** Students use modified versions of an ethical matrix to conduct stakeholder analysis and unpack the implications of design on various groups. They use an ethical matrix to design a new voice assistant for schools *Common Core Standards: RI.6* *CSTA Standards: 2-CS-01*	Explanation of Smart Speakers: https://www.youtube.com/watch?v=o4A3MiLsNJc	40 min
**Text Classification** Students learn the basics of the K Nearest Neighbors algorithm and how it can be used to classify text		20 min
**Exploring Word Analogies** Students use an interactive tool to learn about how word vectors represent language. Students explore bias in large, pretrained datasets and how to mitigate the effects of such bias *CSTA Standards: 2-DA-09, 2-IC-21*	Explore Word Analogies Tool, J. Bazinska and P. Migdal: https://mitmedialab.github.io/arduino-scratch2/word2viz-master/	25 min
**Text Classification and AI Blocks** Students train text classification models and use them to create applications and control robots *CSTA Standards: 2-CS-02*	Programming Platform: https://mitmedialab.github.io/prg-extension-boilerplate/robotafe Programming Tutorial: https://httyr.media.mit.edu/tutorials#h.7kaapo7qg4qv	45 min

### Day 4 Activities

- Teacher guide: https://tinyurl.com/httyr-day4guide
- Slides: https://tinyurl.com/httyr-day4slides

**Table:**

Activity	Links	Time
**Final Project Brainstorming** Students collaboratively generate ideas for final projects that leverage what they learned during the week	Brainstorming Worksheet: https://tinyurl.com/httyr-day4brainstorm	15 min
**Final Project Planning** Students plan the ethical design considerations and technical components they will use in their final projects	Planning Worksheet: https://tinyurl.com/httyr-day4plan	15 min
**Final Project Work Time** Students begin working on their open-ended projects *CSTA Standards: 2-CS-02, 2-AP-12, 2-AP-13, 2-AP-15, 2-AP-17, 2-AP-18*	Programming Platform: https://mitmedialab.github.io/prg-extension-boilerplate/robotafe	105 min

### Day 5 Activities

- Teacher guide: https://tinyurl.com/httyr-day5guide
- Slides: https://tinyurl.com/httyr-day5slides

**Table:**

Activity	Links	Time
**Final Project Work Time** Students wrap up their final projects and create presentations to share their work *CSTA Standards: 2-CS-02, 2-AP-12, 2-AP-13, 2-AP-15, 2-AP-17, 2-AP-18*	Programming Platform: https://mitmedialab.github.io/prg-extension-boilerplate/robotafe Final Project Slide Template: https://tinyurl.com/httyr-day5slide	90 min
**Class Showcase** Students present their final projects to a general audience of their family and friends *Common Core Standards: SL.4*		45 min

## Appendix 4. Prior Experience Questionnaire

**Table:**

Question	Answer Options
Have you heard of AI before? (Creative, HTTYR)	• Yes • No • Not sure
Which of these pieces of technology have you used before?	YouTube, Google Search, Twitter, Email, Amazon Alexa, Netflix, Facebook, Snapchat, Spotify, Cortana, Google Home, Instagram, Lego Mindstorms, Cozmo robot, Tablet, Scratch, Gaming System, TikTok
On a scale of 1 to 5, how much is technology a part of your life? (HTTYR)	• Not at all (1) • Quite a lot (5)

## Appendix 5. AI Perception Questionnaire

**Table:**

Question	Answer Options
How would you describe AI to your friends?	Open ended
Select all of the words that you think describe AI:	Creative, Intelligent, Funny, Original, Fair, Unfair, Collaborative, Tool, Helpful, Harmful, Exciting, Scary
Which of these do you agree with?	• AI will take over jobs • AI will make jobs easier
Which of these do you think AI can do?	• Find the square root of pi • Recognize your face • Create music • Bake a cake • Style your hair • Make a painting • Hit a baseball
Do you think that the following technologies have Artificial Intelligence? (HTTYR)	Google Search, Printer, Video Call App, Game Consoles, YouTube, Voice Assistants, System, TikTok

## Appendix 6. Self Perception Questionnaire

**Table:**

Question	Answer Options
How much do the following statements describe you? • I want to work with computers and technology • I usually find computers and technology confusing • I don’t know a lot about computers and technology • I like to learn about computers and technology • Some of my friends or family work with computers or technology • I would not like a job with computers and technology • I am good with computers and technology	• Not at all (1) • Not really (2) • Unsure (3) • Somewhat (4) • Quite a lot (5)
Which of the following do you agree with the most?	• AI is smarter than me • AI and I are equally smart • I am smarter than AI
Which of these do you agree with?	• I can control AI • AI has a mind of its own • Both • Neither
Which of these do you agree with?	• AI can help me make things • I can help AI make things • Both • Neither

## Footnotes

1 Artificial Intelligence in Education, https://www.iste.org/areas-of-focus/AI-in-education

2 Slice of ML by Google, https://sliceofml.withgoogle.com/

3 Machine Learning 4 Kids by Dale Lane, https://machinelearningforkids.co.uk/

4 ECraft2Learn Project by Ken Kahn, https://ecraft2learn.github.io/ai/

5 Machine Learning for Artists by Gene Kogan, https://ml4a.github.io/

6 Computer Visions: Generative Machine Learning Tools for Creative Applications course at MIT, http://visions.media.mit.edu/

7 Artists and Machine Intelligence by Google, https://ami.withgoogle.com/

8 Creative Machine Learning for Design course at MIT, https://architecture.mit.edu/subject/spring-2020-4453

9 Machine Learning 4 Kids by Dale Lane, https://machinelearningforkids.co.uk/

10 *Dancing with AI* Programming platform, https://mitmedialab.github.io/prg-extension-boilerplate/create)

11 Scratch Github Repository by the Lifelong Kindergarten Group at the MIT Media Lab, https://github.com/LLK/

12 *How to Train Your Robot* programming platform, https://mitmedialab.github.io/prg-extension-boilerplate/robotafe

13 Scratch Github Repository by the Lifelong Kindergarten Group at the MIT Media Lab, https://github.com/LLK/

14 Yahboom Tiny:bit robot car for micro:bit, https://category.yahboom.net/products/tinybit?variant=28480989724756

15 Improving Basic Programs Operated by Local Educational Agencies (Title I, Part A), https://www2.ed.gov/programs/titleiparta/index.html

16 All participant names have been changed to preserve their privacy.
